# Stability of sensorimotor network sculpts the dynamic repertoire of resting state over lifespan

**DOI:** 10.1093/cercor/bhac133

**Published:** 2022-04-04

**Authors:** Nisha Chetana Sastry, Dipanjan Roy, Arpan Banerjee

**Affiliations:** Cognitive Brain Dynamics Laboratory, National Brain Research Centre, NH 8, Manesar, Gurgaon 122052, India; School of Artificial Intelligence & Data Science, Centre for Brain Science & Applications, Indian Institute of Technology, Jodhpur NH 62, Surpura Bypass Rd, Karwar, Rajasthan 342030, India; Cognitive Brain Dynamics Laboratory, National Brain Research Centre, NH 8, Manesar, Gurgaon 122052, India

**Keywords:** dynamic functional connectivity, healthy aging, resting-state networks, temporal stability, Mahalanobis distance

## Abstract

Temporally stable patterns of neural coordination among distributed brain regions are crucial for survival. Recently, many studies highlight association between healthy aging and modifications in organization of functional brain networks, across various time-scales. Nonetheless, quantitative characterization of temporal stability of functional brain networks across healthy aging remains unexplored. This study introduces a data-driven unsupervised approach to capture high-dimensional dynamic functional connectivity (dFC) via low-dimensional patterns and subsequent estimation of temporal stability using quantitative metrics. Healthy aging related changes in temporal stability of dFC were characterized across resting-state, movie-viewing, and sensorimotor tasks (SMT) on a large (*n* = 645) healthy aging dataset (18–88 years). Prominent results reveal that (1) whole-brain temporal dynamics of dFC movie-watching task is closer to resting-state than to SMT with an overall trend of highest temporal stability observed during SMT followed by movie-watching and resting-state, invariant across lifespan aging, (2) in both tasks conditions stability of neurocognitive networks in young adults is higher than older adults, and (3) temporal stability of whole brain resting-state follows a U-shaped curve along lifespan—a pattern shared by sensorimotor network stability indicating their deeper relationship. Overall, the results can be applied generally for studying cohorts of neurological disorders using neuroimaging tools.

## Introduction

Aging is typically associated with substantial structural and functional modifications of the brain. Significant number of studies, which have investigated age-related modifications in functional networks using static functional connectivity, reveal an overall increase in between-network connectivity and decrease in within network connectivity in older adults (Damoiseaux et al. 2008) (Betzel et al. 2014) (Cao et al. 2014) (Chen et al. 2021). Recently, dynamic functional connectivity (dFC) has emerged as a major topic in the resting-state BOLD functional magnetic resonance imaging (fMRI) literature. In spite of inherent limitations (Preti et al. 2017), dFC captures the fluctuations in temporal scale of minutes that contain meaningful information (Hutchison et al. 2013). While accounting for these fluctuations maybe important for understanding the itinerant nature of slow neuronal dynamics, stable representation of information of neural activity and corresponding stability of FC patterns over time is crucial for survival (Li et al. 2019). Secondly, what are the key contributors that shapes stability of FC patterns in ongoing brain dynamics is a vital issue that needs resolution.

Recent evidence suggests FC stability increases with motor learning (Yu et al. 2020), was significantly higher in patients with major depressive disorder (Demirtas et al. 2016), decreases in patients of schizophrenia and their siblings (Guo et al. 2017). Previous studies exploring temporal dynamics of FC have tried to investigate the stability by calculating the correlation between FC matrices computed from successive temporal windows (Hansen et al. 2015), characterizing variability of the functional connectivity profile of a given region across time (Zhang et al. 2016; Guo et al. 2017), by estimating voxel level dFC maps using Kendall’s coefficient of concordance with time windows as raters (Li et al. 2019), by estimating the standard deviation of global modularity averaged across all timepoints and all participants (Hilger et al. 2019).

Further, studies have also explored modifications in temporal stability of functional architecture in resting state of healthy control and patients with psychiatric disorders, and different battery of tasks. Zhang and colleagues showed disorder specific (ADHD, schizophrenia, autism spectrum disorder) variability modifications in functional architecture of default mode network (DMN), visual and subcortical regions of the brain (Zhang et al. 2016). Increased functional stability in high-order visual regions during naturalistic movie watching task was identified (Li et al. 2019), but these studies are limited to stability of FC of a given region.

The temporal stability of functional architecture is shown to influence the relationship between resting state and task-related brain dynamics as well (Li et al. 2019). Spontaneous brain activity during rest is not random and shows specific spatio-temporal organization in state space (Deco et al. 2011). Deco and Jirsa (2012) speculated the existence of a multistable attractor landscape that describes the dynamic repertoire of resting state. Brain resides in a specific attractor state defined by a certain FC pattern according to the cognitive demands of the task (Fedorenko and Thompson-Schill 2014; Pillai and Jirsa 2017). An overall increase in FC stability has been reported in the presence of the task (Gonzalez-Castillo and Bandettini 2018). Thus, we hypothesized, unsupervised dFC characterization will reveal task-specific dFC stability patterns that are local in time, i.e. limited, abated spread of stability patterns, whereas for the resting-state dFC patterns, these functional states are composed of nonlocal correlations in time, with more global, widespread stability patterns.

Although previous studies have explored the association between dFC and age (Viviano et al. 2017; Chen et al. 2017; Xia et al. 2019), how the stability of functional architecture modifies across lifespan aging remains an open question. Further, the age-related changes in temporal stability across resting-state networks (RSN) need to characterized.

The aim of the present study is 2-fold: (1) to precisely characterize the stability of whole-brain dFC patterns during task and rest for a cross-sectional population over human adult lifespan (18–88 years) using a novel unsupervised approach (2) to identify the contributors (subnetworks) to dFC temporal stability in resting human brain and how they organize over lifespan. This manuscript is organized as follows. First, we estimate BOLD phase coherence over time (Glerean et al. 2012), which was used as a measure of dFC for rest and task. Next, we proceed with unsupervised characterization of dFC subspaces involved in task and rest. Subsequently, the temporal stability of dFC subspaces was computed using 2 different measures—angular separation and the Mahalanobis distance (Mahalanobis 1930; Shen et al. 2010). Finally, we analyze the temporal stability of dFC to draw critical insights about age-associated differences to task and rest using a large human cohort (*n* = 645) of healthy aging (Shafto et al. 2014).

## Methods

### Data sources and participants

The data were collected as part of stage 2 of the Cambridge Centre for Aging and Neuroscience (CamCAN) project (available at http://www.mrc-cbu. cam.ac.uk/datasets/camcan) (Taylor et al. 2017; Shafto et al. 2014). The CamCAN is a large-scale multimodal, cross-sectional, population-based study. The database includes raw and preprocessed structural MRI, resting state and active tasks using fMRI and magnetoencephalogram (MEG), behavioral scores, demographic and neuropsychological data. From 3,000 participants of stage 1, a subset of approximately 700 participants who were cognitively healthy (Mini-Mental State Examination (MMSE) score > 25), with no past or current treatment for drug abuse or usage, met hearing threshold greater than 35 dB at 1,000 Hz in both ears, had at least a corrected near vision of 20/100 with both eyes and could speak English language (native English speaker or bilingual English from birth) were eligible for MRI scanning. They were home interviewed and recruited to stage 2. The study was in compliance with the Helsinki Declaration and was approved by the Cambridgeshire 2 Research Ethics Committee. The fMRI data from resting state and task periods (naturalistic movie watching and sensorimotor task [SMT]) were used in the present study.

### Data acquisition and experimental paradigm

The fMRI data were collected at MRC Cognition and Brain Sciences Unit, on a 3 T Siemens TIM Trio scanner with a 32-channel head coil, the head movement was restricted with the aid of memory foam cushions. For the tasks, the instructions and visual stimuli were back projected onto the screen, auditory stimuli were presented via MR-compatible Etymotics headphones and manual responses from the participants made with the right hand were recorded using an MR-compatible button box (Taylor et al. 2017). The fMRI data for eyes-closed resting state and SMT were acquired using echo-planar imaging (EPI) sequence, consisted of 261 volumes, each volume with 32 axial slices (slice thickness 3.7 mm, interslice gap 20% for whole-brain coverage) acquired in descending order, TR 1,970 ms, TE 30 ms, voxel-size 3 mm × 3 mm × 4.44 mm, flip angle 78°, field-of-view 192 mm × 192 mm, bandwidth 2,232 Hz/Px. The duration of both the scans was 8 min 40 s. The fMRI data for the naturalistic movie watching task were acquired using multiecho EPI sequence, consisting of 193 volumes of 32 axial slices each (slice thickness 3.7 mm, interslice gap 20% for whole brain coverage) acquired in descending order, TR 2,470 ms, TE [9,4,21.2,33,45,57] ms, voxel-size 3 mm × 3 mm × 4.44 mm, flip angle 78°, field-of-view 192 mm × 192 mm, bandwidth 2,520 Hz/Px. The duration of the scan was 8 min 13 s. A detailed description of data acquisition parameters can be found in (https://camcan-archive.mrc-cbu.cam.ac.uk/dataaccess/) (Taylor et al. 2017).

The task-induced Blood Oxygen Level dependent (BOLD) data from the naturalistic movie watching task were acquired from participants, who watched 8 min of narrative preserved, condensed, black and white version of Alfred Hitchcock’s television drama “Bang! You’re Dead.” The participants were not aware of the title of the movie but were instructed to pay attention to the movie. In the SMT, the trials consisted of a binaural tone simulation at either 300, 600, or 1,200 Hz and bilateral black and white checkerboard. The participants were asked to button press with their right index finger if they hear or see any stimuli. More details about the task paradigm have been presented here (Shafto et al. 2014; Taylor et al. 2017).

### Data preprocessing

The fMRI data for each functional run (resting state, movie watching, and SMT) were unwarped using field-map images, realigned to correct for motion and, slice-time corrected. EPI data were coregistered to the T1 image, transformed to montreal neurological institute (MNI) space using the warps and affine transformation from structural image (estimated using DARTEL). For region of interest (ROI) analysis, mean regional BOLD time series were estimated in 116 parcellated brain areas of Anatomical Automatic Labelling atlas (AAL) (Tzourio-Mazoyer et al. 2002) (available at http://www.gin.cnrs.fr/tools/aal). Preprocessed data were provided by Cam-CAN research consortium. Detailed overview of preprocessing pipeline can be found in Taylor et al. (2017). In order to capture the pattern of temporal stability over lifespan (e.g.: of the “Entropy” metric), we divided the whole dataset of *n* = 645 participants into nonoverlapping bins of 5 years starting from 18 years. On the other hand, to gather accurate insights in each stage of the adult lifespan, we divided the whole data of *n* = 645 participants into 3 cohorts, young adults with an age range 18–40 years (50.27% female, mean age = 31.21 ± 6.06 years), middle adults with an age range 41–60 years (52.23% female, mean age = 50.49 ± 5.70 years), old adults with an age range 61–88 years (49.92% female, mean age = 73.72 ± 7.20 years). Each participant’s BOLD time series in the resting state, naturalistic movie watching, and SMTs were extracted.

### Data analysis

#### Characterization of dynamic functional connectivity

**Fig. 1:**
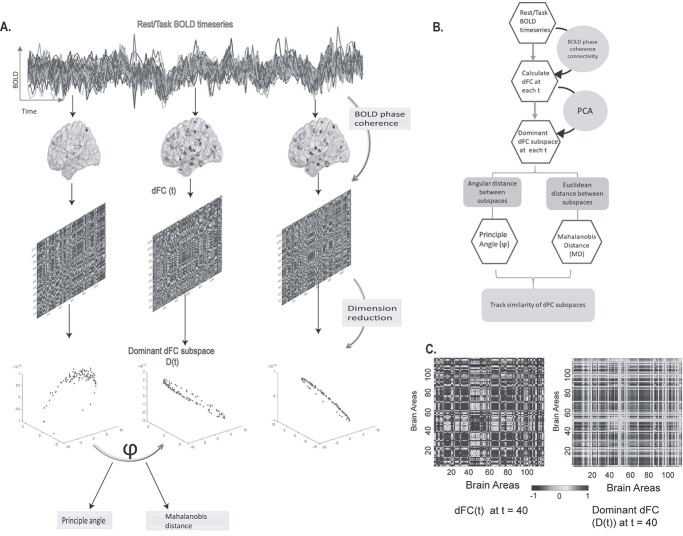
Brief overview of the unsupervised approach (A). The schematic diagram shows how the temporal stability of dFC subspaces are computed. Dominant dFC subspace, at each time point, is estimated using the first 3 principal components of dFC(*t*), which was computed using the measure of BOLD phase coherence. The similarity between dFC subspaces are calculated using angular distance (principal angle) and Mahalanobis distance (Euclidean distance). If the dominant dFC subspaces are similar for extended timepoints, then they are considered to be stable. (B). A flowchart representation of the method (C). Matrix representation of dFC patterns (dFC(*t*)) and reduced dominant dFC patterns (*D*(*t*)) at *t* = 40.

Time-resolved dFC was estimated, for each individual, using BOLD phase coherence (Fig. 1A; Glerean et al. 2012; Ponce-Alvarez et al. 2015; Deco and Kringelbach 2016; Cabral et al. 2017), which resulted in a matrix with size *N* x *N* x *T*, where *n* = 116 is the number of brain regions defined by AAL atlas, *T* is the total number of time points (*T* = 261 for resting state and SMT, *T* = 193 for naturalistic movie watching task). We chose BOLD phase coherence instead of computing correlation over a sliding window to calculate dFC, because BOLD phase coherence is an instantaneous measure with maximum temporal resolution (Glerean et al. 2012). BOLD phase coherence does not require time-windowed averaging, which generates biased estimates if the window length is short and reduces temporal resolution if the window length is longer (Glerean et al. 2012).

First, the instantaneous phases $\theta (n,t)$of the BOLD time series for all the brain regions, $n$, was computed using Hilbert transform. The real-valued modulated BOLD signal $s(t)$ is expressed as an analytical signal in the complex plane as:

(1) \begin{equation*} z(t)={z}_r(t)+j{z}_i(t)=s(t)+j\ HT\left[s(t)\right] \end{equation*}

where (HT [*]) represents the Hilbert transform. The instantaneous phase $\theta\ (t)$is computed as follows:

(2) \begin{equation*} \theta (t)=\angle z(t)=\arctan \frac{z_i(t)}{z_r(t)} = \arctan \frac{HT\ \left[s(t)\right]}{s(t)} \end{equation*}

Given the phases of the BOLD time series, phase coherence, i.e. $\mathrm{dFC}\ (n,p,t)$ for brain regions, $n$ and $p$at time $t$is computed as:

(3) \begin{equation*} \mathrm{dFC}\ \left(n,p,t\right)=\cos \left(\theta \left(n,t\right)-\theta \left(p,t\right)\right) \end{equation*}

when the phases of BOLD signals,$\theta (n,t)$, $\theta (p,t)$ of the brain regions $n,p$ are synchronized, $dFC(n,p,t)$(ranges from −1 to 1) is close 1, when the phases from the BOLD signals of brain regions $n,p$are orthogonal $\mathrm{dFC}(n,p,t)$is close to 0. Since the phases are undirected, $\mathrm{dFC}(n,p,t)$is symmetric along the diagonal.

In addition to this, to check for reliability, we compute dFC using a sliding-window approach (Hutchison et al. 2013) with nonoverlapping, gaussian windows, varying the window length (10, 20, 30 time points) (Supplementary Information—S1, S2, and S3).

#### Extracting dominant dynamic functional connectivity

Principal component analysis (PCA) was applied to participant-wise $\mathrm{dFC}\ (n,p,t)$matrix of size $N\ \mathrm{X}\ N$ representing the FC between $n$th and $p$th brain area for each time point. PCA is an unsupervised, multivariate dimension reduction method that decomposes the data into a set of orthogonal principal components or leading eigenvectors sorted by their contribution to the overall variance (Friston et al. 1993). Thus, $\mathrm{dFC}\ (n,p,t)$ or simply ${\mathbf{dFC}}_t$ can be expressed as

(4) \begin{equation*} {\mathbf{dFC}}_t={\boldsymbol{V}}^T\boldsymbol{SV} \end{equation*}

where matrix $\boldsymbol{V}$ of size $N\ \mathrm{X}\ N$ are set of eigenvectors, with each column of $\boldsymbol{V}$ of size $1\ \mathrm{X}\ N$ representing orthogonal principal component, and $\boldsymbol{S}$ is the diagonal matrix $$ \\ \left(\begin{array}{ccc}{\lambda}_1& \cdots & 0\\{}\vdots & \ddots & \vdots \\{}0& \cdots & {\lambda}_N\end{array}\right)$$, such that ${\lambda}_1>{\lambda}_2\dots .>{\lambda}_{N.}$

If $k$ is the number of principal components chosen to represent $\mathbf{dFC}$, the corresponding subspace $\boldsymbol{D}(\boldsymbol{n},\boldsymbol{k},\boldsymbol{t})$ or ${\boldsymbol{D}}_{\boldsymbol{t}}$, representative of dominant dFC pattern, can be expressed as

(5) \begin{equation*} \boldsymbol{D}={\overset{\sim }{\boldsymbol{V}}}^{\boldsymbol{T}}\overset{\sim }{\boldsymbol{S}}\overset{\sim }{\boldsymbol{V}} \end{equation*}

where ${\overset{\sim }{\boldsymbol{V}}}^{\boldsymbol{T}}$is a dimensionally reduced matrix of size $N\ \mathrm{X}\ k$,$\overset{\sim }{\boldsymbol{S}}$is a diagonal matrix $$\left(\begin{array}{ccc}{\lambda}_1& \cdots & 0\\{}\vdots & \ddots & \vdots \\{}0& \cdots & {\lambda}_k\end{array}\right)$$. In this study, we chose *k* = 3 because for all participants at least 99% variance in $\mathbf{dFC}$ matrix is captured by the 3 leading eigenvectors **(S 4)**. The dimension of $\mathbf{dFC}(\boldsymbol{n},\boldsymbol{p},\boldsymbol{t})$ has been reduced to $\boldsymbol{D}(\boldsymbol{n},\boldsymbol{k},\boldsymbol{t})$**.** Since, the dFC(*t*) matrices are symmetrical, several studies compare only the upper triangular elements (Cabral et al. 2017). In our study, we use an alternative method, where we consider the first 3 leading eigen vectors, forming a reduced 3D dominant dFC subspace **(*D*(*t*))** of each **dFC (*t*)**. Compared to considering all (upper triangular) the elements of dFC(t), this method reduces the dimensionality of the of the data while still explaining almost 99% of the variance (Supplementary S4). This method of estimating dFC(*t*) also bypasses the use of sliding window approach to estimate dFC.

#### Computation of stability of dynamic functional architecture

We seek to characterize the temporal stability of the dominant subspace $D(n,p,t)$ (or referred to as simply ${\boldsymbol{D}}_t$) by estimating how similar they are across time $t$. To estimate the similarity between dominant dFC configurations, we introduce 2 types of distance measures successive dFC subspaces, (1) angular distance and (2) normalized Euclidean distance (Fig. 1B). We define angular distance as the principal angle between the dFC subspaces from different time points, given by the following equation:

(6) \begin{equation*} \phi \left({t}_x,{t}_y\right)=\angle \left({\boldsymbol{D}}_{t_x},{\boldsymbol{D}}_{t_y}\right) \end{equation*}

where each entry in the time × time temporal stability matrix, $\phi ({t}_x,{t}_y)$ is the principal angle between the 2 $N\times k$dimensional subspaces at ${t}_x$ and ${t}_y$ (Björck and Golub 1973; Banerjee et al. 2012). The principal angle ranges between 0 (low angular distance) to *π*/2 (high angular distance).

For each individual, we calculate the angular distance between dominant dFC subspaces at ${t}_x$ and ${t}_y$, by estimating the principal angle between them. The low principal angle between dominant dFC subspaces means that their dFC configurations are very similar. On the contrary, the high principal angle between dominant dFC subspaces means that their dFC configurations are dissimilar.

We define the normalized Euclidean distance between dominant dFC subspaces by the Mahalanobis distance. Mahalanobis distance measures the distance between points in space 1 from space 2 with the following equation:

(7) \begin{equation*} {M}^2={\left({\boldsymbol{D}}_{t_x}-{\boldsymbol{D}}_{t_y}\right)}^T{C}^{-1}\left({\boldsymbol{D}}_{t_x}-{\boldsymbol{D}}_{t_y}\right) \end{equation*}

where ${M}^2$ is the distance between each entry of ${\boldsymbol{D}}_{t_x}$and ${\boldsymbol{D}}_{t_y}$. For each timepoint, Mahalanobis distance was calculated between each ROI in the reduced dominant dFC subspace ${\boldsymbol{D}}_{t_y}$and whole-brain subspace ${\boldsymbol{D}}_{t_x}$. Every ROI in ${\boldsymbol{D}}_{t_y}$ (point P) has Mahalanobis distance estimated with respect to subspace ${\boldsymbol{D}}_{t_x}$ (distribution D). Subsequently, for each individual, we estimate the time × time *temporal stability matrix*, where each entry is the Mahalanobis distance ($M$ranges between 0.5 and 2.5), averaged across all brain parcels. Low $M$ means that dominant dFC subspaces are similar, high $M$ means that the dFC subspaces are dissimilar. Figure 1C illustrates the matrix representation of dFC patterns **(dFC(*t*))** and reduced Dominant dFC patterns **(*D*(*t*))**.

#### Quantifying complexity of temporal stability matrices

*Entropy*

To evaluate the informational content of *temporal stability matrices* we evaluated the entropy, for all 3 categories, rest, movie viewing, and SMT in young and old adults. Entropy is defined by the following equation:

(8) \begin{equation*} E=-\sum p\ \log (p) \end{equation*}

where $p$ contains the normalized histogram counts returned from “imhist.m.” “imhist.m” calculates the histogram of *temporal stability matrices,* estimated using reduced ${\mathbf{D}}_{t_x}$and ${\mathbf{D}}_{t_y}$, and returns histogram counts. We calculate entropy of *temporal stability matrices*, where each entry is angular distance or Mahalanobis distance estimated between reduced ${\boldsymbol{D}}_{t_x}$and ${\boldsymbol{D}}_{t_y}$, for each subject and condition. In our formulation, Entropy provides us a measure of distinguishable temporal order that can be interpreted as overall stability of the *temporal stability matrices.*

*Frobenius norm*

Frobenius norm was used to measure the differences between the temporal stability matrices computed for rest and the task conditions. Frobenius norm, also called the Euclidean norm of a matrix, is defined as the square root of the sum of the absolute squares of its elements. Here, we calculate Frobenius norm between temporal stability matrices with the following equation:

(9) \begin{equation*} \left|\left|{x}_F\right|\right|=\sqrt{\sum_{i=1}^T\sum_{j=1}^T{\left|{a}_{ij}-{b}_{ij}\right|}^2} \end{equation*}

where ${a}_{ij}$ and ${b}_{ij}$ are the entries in the temporal dynamic matrices of rest and any of the task conditions respectively (movie watching or sensorimotor). ${x}_F$ is also computed between the 2 tasks.

### *Stochastic characterization of*  $\boldsymbol{dFC}$

The temporal variation of 2 measures, principal angle and Mahalanobis distance between the dominant $\mathrm{dFC}$ subspaces essentially capture the degree of temporal variation in functional network. Principal angular values close to $\frac{\pi }{2}$ or high Mahalanobis distance at a specific time point reflects the reorganization of the functional state itself, whereas angular values closer to zero or low Mahalanobis distance indicates minor deviation from previous time. To understand the underlying stochastic characteristics of these measures, we use auto-regressive (AR) models where present values of $\phi (t)$and $M(t)$ are modeled as a linear weighted sum of values from past $\phi (t-1),\phi (t-2)\dots \phi (t-i)/M(t-1),M(t-2)\dots M(t-i)$.The AR ($\rho)$ process, ${X}_t$ ($\phi (t)\ \mathrm{or}\ M(t)$) is given by the following equation:

(10) \begin{equation*} {X}_t=c+\sum_{i=1}^{\rho }{\varphi}_i{X}_{t-i}+{\varepsilon}_t \end{equation*}

where ${\varphi}_1\dots \dots \dots \dots{\varphi}_{\rho }$ are parameters of the model, $c$ is a constant, ${\varepsilon}_t$ is white noise, and $\rho$ is the lag term or model order. The simplest AR process is AR (0) is essentially a white noise process. In AR (1), the current value is dependent only on its immediately preceding value, and hence captures a Markovian process. Optimal model of an AR process can be computed using the Akaike information criterion (AIC), which is expressed as

(11) \begin{equation*} \mathrm{AIC}\left(\rho \right)=-2L+2\rho \end{equation*}

where $L$ is the likelihood function computed by summing up over the mean-squared error for an AR model of order $\rho$ (Wagenmaker and Farrell 2004; Akaike 1974). Optimal model order can be selected at a value of $\rho$, where AIC is minimum. We varied the model order ($\rho$) from 0 to 100 and use the first minimal AIC value to select the best AR ($\rho$), model. If the model order is found to be greater than 1, the underlying process is considered non-Markovian.

## Results

### Dynamic functional connectivity patterns during rest, continuous naturalistic movie watching, and discrete sensorimotor task

**Fig. 2:**
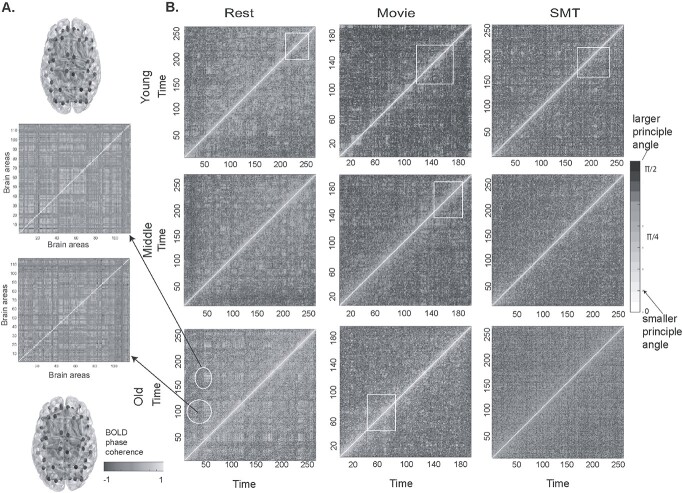
Using angular distance to characterize temporal stability matrices across age (A) dFC matrices estimated using BOLD phase coherence. (B) Time × time temporal stability matrix of resting state, naturalistic movie watching task and discrete, SMT for young, middle, and old adults. Each entry in the matrix is the principal angle $\phi \Big({t}_x,{t}_y\Big)$ between dominant dFC subspaces at ${t}_x$ and ${t}_y$. The principal angle ranges between 0 (low angular distance) to *π*/2 (high angular distance). Resting state, in young, middle, and old adults, has shorter-lived, global spread of patterns of temporal stability. On the contrary, both the tasks have a longer-lived, local spread of patterns of stability (indicated by arrows and rectangular boxes).

We computed the $\mathrm{dFC}$ from parcellated BOLD time series of resting state, naturalistic movie watching task where the participants watched and listened to an excerpt from Alfred Hitchcock’s “Bang! You’re Dead,” and a SMT where participants responded by a button press to either a visual or an auditory stimulus from the Cam-CAN dataset (details in Methods). Figure 2A represents dFC obtained using BOLD phase coherence connectivity in resting state. To capture the pattern of temporal stability over lifespan we divided the whole dataset of *n* = 645 subjects into nonoverlapping bins of 5 years starting from 18 to 88 years. Subsequently, to gather insights at each stage of adult lifespan we have divided the total *n* = 645 subjects into 3 age groups —young, middle, and old with sufficient number of participants (>180) in each category. We report the results of the analysis on young adults (age range 18–40) in this section.

**Fig. 3:**
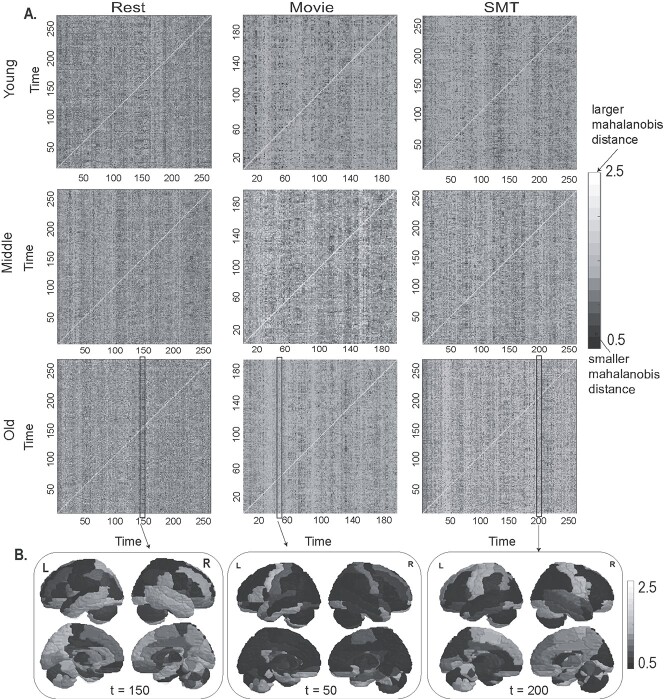
Using Mahalanobis distance to characterize temporal stability matrices across age. (A) Time × time temporal stability matrix of resting state, naturalistic movie watching task, and SMT for young, middle, and old adults, where each entry in the matrix is Mahalanobis $\Big({M}^2\Big({t}_x,{t}_y\Big)\Big)$ distance between the dominant dFC subspaces. Mahalanobis distance between dominant dFC subspaces is low when the dFC configurations are similar. (B) The profile of temporal stability estimated with Mahalanobis distance between dominant dFC subspaces at *t* = 15 and *t* = 150, *t* = 50, *t* = 200 across the brain regions.

Dominant $\mathrm{dFC}$ subspaces were obtained by applying the unsupervised approach of PCA to BOLD time series at each time point, and then reconstructing either the task or rest as the dynamics of a reduced dimensional $\mathrm{dFC}$ subspace. To demonstrate, that the unsupervised characterization of $\mathrm{dFC}$ patterns indeed capture the functional brain network organization, we computed the differences between the temporal stability matrices of rest and the 2 task conditions; first using the measure of principal angle (Fig. 2) and second using the measure of Mahalanobis distance (Fig. 3). Thereafter, other measures of complexity and temporal variability were tested.

#### Using angular distance to characterize temporal stability matrices

**Fig. 4:**
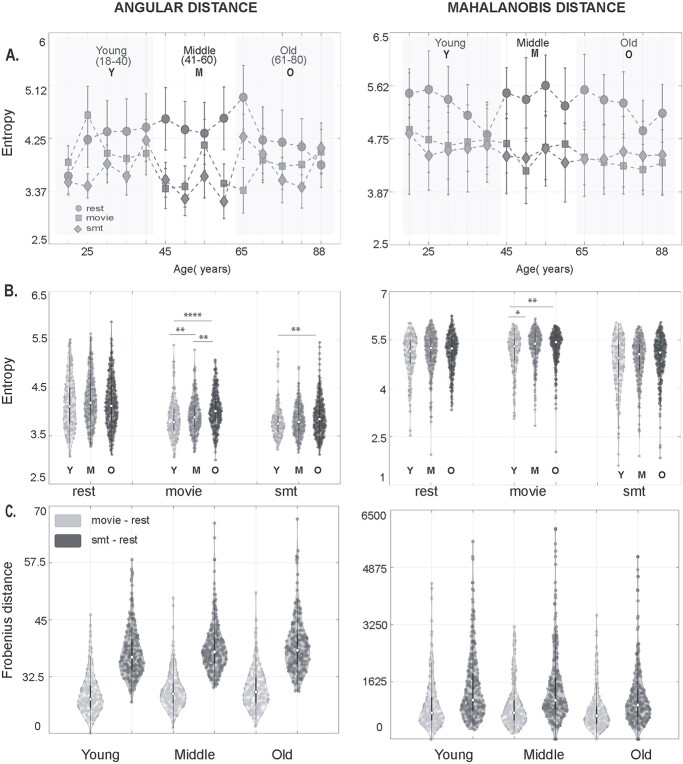
Quantifying complexity of temporal stability matrices across age (A) plots representing entropy of temporal stability matrices of resting state (rest), naturalistic movie watching task (movie), and SMT across lifespan, for angular distance and Mahalanobis distance metric. The subjects were divided into nonoverlapping bins of 5 years starting from 18 years to 88 years (18–20, 21–25, 26–30, 31–35, 36–40, 41–45, 46–50, 51–55, 56–60, 61–65, 66–70, 71–75, 76–80, 81–88). (B) Plots representing entropy of temporal stability matrices of resting state (rest), naturalistic movie watching task (movie) and SMT for young (cyan), middle (blue), and old (pink) adults, for angular distance and Mahalanobis distance metric. Statistically significant differences (uncorrected) are indicated using ^*^($\mathcal{P}\le 0.05)$, ^**^$(\mathcal{P}\le 0.01)$, ^***^$(\mathcal{P}\le 0.01)$, ^***^$(\mathcal{P}\le 0.001)$, ^****^$(\mathcal{P}\le 0.0001)$, ns (not significant). (C) Plots representing distribution of Frobenius distance between temporal stability matrices of resting state, naturalistic movie watching and resting state, SMT for angular distance, and Mahalanobis distance metric, in young, middle and old adults. The violin plots reveal a shorter Frobenius norm between resting state and movie watching task than resting state and SMT in all young, middle, and old adults.

First, we calculate the principal angles among the dominant $\mathrm{dFC}$ subspaces generated across all time points. This resulted in time × time temporal stability matrix, averaged across all subjects, where each entry in the matrix is the angle between dominant $\mathrm{dFC}$ subspaces at ${t}_x$ and ${t}_y$, as shown in Fig. 2B. We consider a dominant $\mathrm{dFC}$ configuration to be stable if the subsequent subspaces are similar in configuration, i.e. less “angular distant” for extended duration of time points. Results shown in Fig. 2B indicate that the resting state has a global spread of shorter-lived, repeated patterns of stability than both tasks. On the contrary, both the task cohorts, passive movie watching, and SMT, showed a local spread of, longer-lived stability patterns suggesting that local temporal stability of functionally connected networks are higher in the task than in resting state. To quantify these observations, we calculate the entropy of temporal stability matrices of resting state, movie watching task, and SMT. The plots in Fig. 4A, which represent entropy of temporal stability matrices of 3 categories of rest and task across lifespan, overall, report resting state to have the highest entropy, followed by movie watching task and SMT. We also calculate entropy of temporal stability matrices of young, middle, and old age categories across resting state and both task cohorts (see Supplementary S5). Wilcoxon sign rank test revealed significant differences in entropy of temporal dynamic matrices of rest and task cohorts (details reported in Supplementary Material). Further, to analyze how similar temporal stability matrices across rest and tasks are, we calculate the Frobenius norm as shown in Fig. 4C. The results reveal a shorter Frobenius norm between the temporal dynamic matrices of the resting state and movie watching task, than the resting state and SMT.

#### Using Mahalanobis distance to characterize temporal stability matrices

Alternatively, we evaluate the temporal stability of $\mathrm{dFC}$, by estimating Mahalanobis distance, that resulted in a time × time temporal stability matrix. Each entry of this matrix is the Mahalanobis distance between dominant dFC subspaces. Results, as shown in Fig. 3A and B, reveal global, shorter-lived repeated patterns of temporal stability in resting state and local, longer-lived temporal stability patterns in both the tasks. The entropy results across lifespan (Fig. 4A) reveal an overall high entropy in the resting state, followed by movie watching task and SMT. We repeat the Frobenius norm analysis, which produced similar results as the angular distance metric, as shown in Fig. 4C.

### Unsupervised characterization of $\mathbf{dFC}$ across healthy lifespan aging

Next, we have included 3 age cohorts, young, middle, and old adults from the Cam-CAN dataset and carried out unsupervised characterization of dFC using participant’s resting state, movie watching, and SMT data to identify age associated alterations in temporal stability of dominant $\mathrm{dFC}$ subspaces.

#### *Using angular distance to quantify temporal stability differences in*  $\mathrm{dFC}$  *across healthy aging*

The time × time temporal stability matrix was computed for the middle (age range 41–60 years) and older cohort (age range 61–88 years) and compared with that of younger cohort computed in Section 3.1. A global spread of shorter duration of temporal stability patterns was observed in resting state and local spread of longer duration temporal stability patterns was observed in the task, in all young, middle, and old adults (see Fig. 2B). Further, entropy analysis revealed (see Fig. 4B), in movie watching and SMT, a peak entropy in older adults, followed by middle and young adults. Whereas in resting state, we observed peak entropy in middle adults, followed by older adults and their younger counterparts. The distributions were nonparametric (normality check was done with Jarque-Bera test and D’Agostino-Pearson omnibus test), Wilcoxon rank sum test revealed significant differences in entropy values between young and middle adults (*P* = 0.0073), middle and old adults (*P* = 0.0017) and, young and old adults (*P* = 2.24e-08) in movie watching task and young and old adults (*P* = 0.0019) in SMT. The Frobenius norm analysis as shown in Fig. 4C also revealed a similar trend in young and old adults, i.e. shorter Frobenius norm between resting state and movie watching task than resting state and SMT

#### *Using Mahalanobis distance to quantify temporal stability of*  $dFC$  *across healthy aging*

Mahalanobis distance between dominant dFC subspaces showed patterns similar to principal angle in young, middle and elderly. Further, we calculate entropy as shown in Fig. 4B, of temporal stability matrices of each age category, in both rest and task conditions. The results indicate peak entropy in older adults, followed by middle and young adults in movie watching and SMT and, peak entropy in middle adults, followed by old and young adults in resting state, a similar trend as the angular distance metric. The distributions were nonparametric (normality check was done with Jarque-Bera test and D’Agostino-Pearson omnibus test). Wilcoxon rank sum test revealed statistical significance between the entropy of temporal stability matrices of young and middle adults (*P* = 0.0176), young and older adults (*P* = 0.0063) in movie watching task. Frobenius norm analysis as shown in Fig. 4C revealed a shorter Frobenius norm between resting state and movie watching task than resting state and SMT.

### Age related changes in temporal stability of dFC in resting state brain networks

**Fig. 5:**
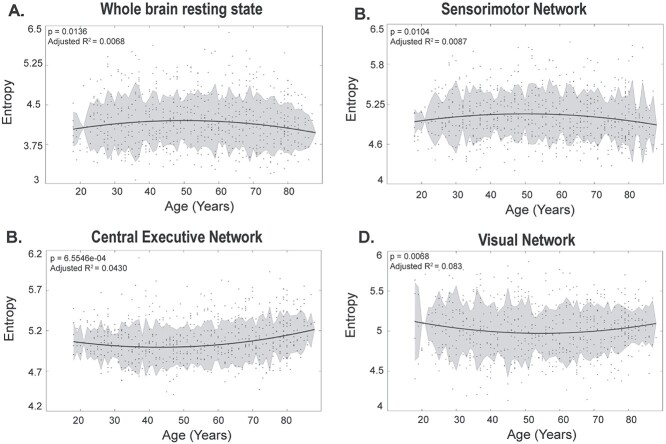
Age-related changes in temporal stability of dFC of whole-brain resting state (A), SM (B), CEN (C), and visual network (D) estimated with angular distance metric. Only those networks with significant modifications in temporal stability with age are shown. Statistically significant differences (uncorrected) are indicated using ^*^($\mathcal{P}\le 0.05)$, ^**^$(\mathcal{P}\le 0.01)$, ^***^$(\mathcal{P}\le 0.01)$, ^***^$(\mathcal{P}\le 0.001)$, ^****^$(\mathcal{P}\le 0.0001)$, ns (not significant).

**Fig. 6:**
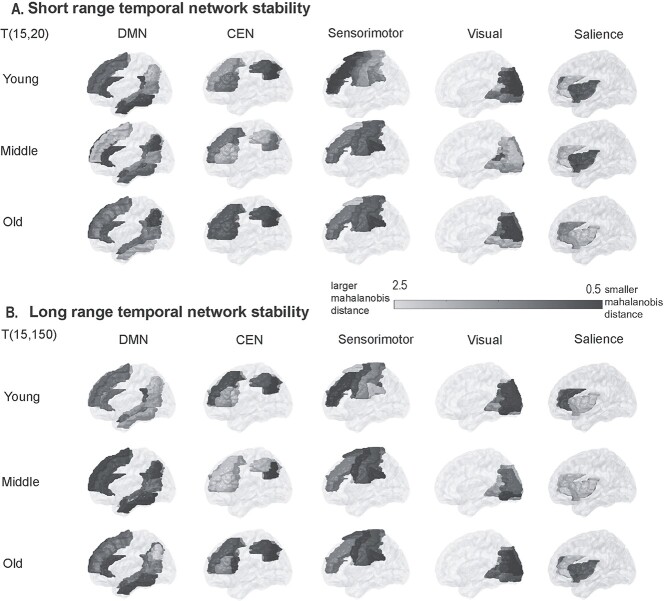
Spatial profile of Mahalanobis distance across RSNs (A) short-range temporal stability—Mahalanobis distance between dominant dFC subspaces of DMN, CEN, SM, salience, and visual brain networks at *t* = 15 and *t* = 20. (B) Long-range temporal stability—Mahalanobis distance between dominant dFC subspaces of DMN, CEN, SM, salience, and visual brain networks at *t* = 15 and *t* = 150.

Next, we apply our unsupervised approach of PCA to BOLD time series of 5 resting-state brain networks and at each time point estimate dominant $\mathrm{dFC}$ subspaces specific to the 5 resting-state brain networks. DMN regions were determined primarily according to (Fox et al. 2005) and ROIs in the AAL atlas specific to DMN were selected according to Wang et al. (2012). We selected ROIs specific to sensorimotor (SM) and visual networks according to (Figueroa-Jimenez et al. 2020) and ROIs specific to central executive network (CEN) were selected according to (Oliver et al. 2019). We selected ROIs in AAL atlas specific to salience network comprising of dorsal anterior cingulate cortex and anterior insula. Further, we estimate entropy to capture the temporal stability of dominant dFC subspaces of whole brain resting state and dominant dFC subspaces specific to 5 resting-state brain networks (DMN, CEN, SM, salience, and visual) using angular distance and Mahalanobis distance, across lifespan aging. We report only significant modifications in temporal stability of dFC subspaces with age in Fig. 5 (we report others in Supplementary S6) Regression analysis revealed a “U” shaped trend for temporal stability of dFC subspaces of whole-brain resting state, sensorimotor, central executive, and visual network with age. We found significant increase in temporal stability of whole brain resting state in older adults (Fig. 5A). Of the 5 resting-state brain networks, the temporal stability of sensorimotor network (SM) exhibited significant increase in older adults (Fig. 5B), similar to whole brain resting state. The temporal stability of CEN (Fig. 5C) and, visual network (Fig. 5D) demonstrated significant decrease with age. We found no significant modifications in temporal stability of dFC with Mahalanobis distance metric. We report results estimated using Mahalanobis distance metric in Supplementary S7. Detailed overview of the terms included in the regression analysis is shown in Supplementary S8. The profile of temporal stability, estimated with Mahalanobis distance across brain regions is shown in Fig. 6. We report 2 examples for young, middle, and older adults—(1) short-range temporal stability, i.e. Mahalanobis distance between dominant dFC subspaces of DMN, CEN, SM, salience, and visual networks at *t* = 15 and *t* = 20 (see Fig. 6A) and (2) long-range temporal stability, i.e. Mahalanobis distance between dominant dFC subspaces of DMN, CEN, SM, salience, and visual networks at *t* = 15 and *t* = 150 (see Fig. 6B). In summary, short- and long-range temporal stability was assessed empirically by comparing time points that were 5 and 135 data points apart, respectively.

### Stochastic characterization of $\mathbf{dFC}$

**Fig. 7:**
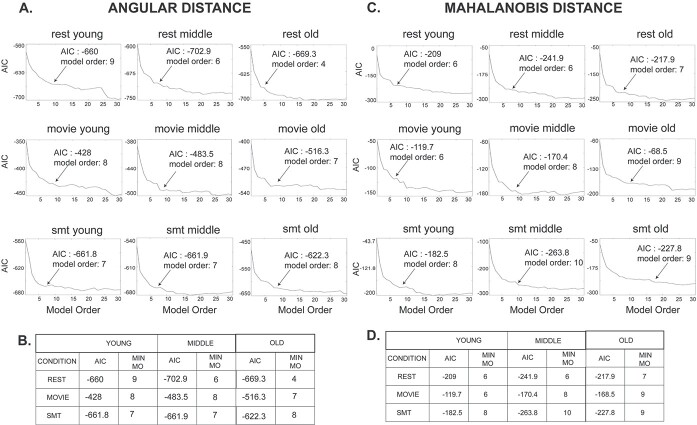
Stochastic characterization of dFC (A) stochastic modeling of principal angle, $\phi\ (t)$ as autoregressive, AR $(\rho)$ process. The model order ($\rho)$ was varied from 0 to 100. The plot represents AIC values corresponding to the model order. Inset shows the first minima of the AIC value and its corresponding model order. (B) Table shows first minimal AIC value and its corresponding model order of $\phi\ (t)$ for all the categories (C) stochastic modeling of Mahalanobis distance, $M\ (t)$as AR $(\rho)$ process. The model order ($\rho)$ was varied from 0 to 100. The plot represents AIC values corresponding to the model order. Inset shows the first minima of the AIC value and its corresponding model order. (D) Table shows first minimal AIC value and its corresponding model order of $M\ (t)$for all the categories.

We examined the stochastic structure of $\mathrm{dFC}$ evolution by investigating the principal angle $\phi\ (t)$ and Mahalanobis distance $M\ (t)$as functions of time. $\phi\ (t)$ and $M(t)$ are modeled as auto-regressive or AR ($\rho)$ process. The optimal model order was taken to be at the value that yields first lowest AIC. The results from this analysis shown in Fig. 7A and B reveal the best fit model that explains $\phi\ (t)$ has a model order ρ $\ge 4$, i.e. the results suggest $\phi\ (t)$ of resting state, movie watching task and SMT, in both young and old adults, is neither random (ρ$\ne 0)$ nor Markovian (ρ$\ne 1)$ in nature, and is dependent on at least 4 immediately preceding values of $\phi .$ For $M(t)$, as shown in Fig. 7C and D both resting state and tasks have the optimum model order $\rho \ge 6$, suggesting $M(t)$ is neither random (ρ$\ne 0)$ nor Markovian (ρ$\ne 1)$ in both young and old adults.

## Discussion

The functional architecture of the brain is dynamic and changes on a minute temporal scale during resting state and task (Hutchison et al. 2013; Gonzalez-Castillo et al. 2015; Gonzalez-Castillo and Bandettini 2018; Bolton et al. 2020). While previous studies have explored flexibility (Zhang et al. 2016; Yin et al. 2016) and temporal variability (Zhang et al. 2016; Li et al. 2019) of the functional architecture of a specific region, we propose a novel unsupervised method, that captures the stability of whole-brain functional architecture on a minute temporal scale. First, we apply the data-driven unsupervised approach to characterize the high-dimensional dFC into lower dimensional patterns by identifying temporally similar dominant FC configurations. Subsequently, using 2 different measures—principal angle and Mahalanobis distance applied on dFCs extracted across time, we capture the stability of dFC through the *temporal stability matrices* that could be used to draw critical insights about underlying functional brain states. For empirical validation, we explored modifications in temporal stability matrices of whole-brain FC during a continuous, naturalistic movie watching task and discrete, goal oriented SMT and showed that, in contrast to resting state, stability increased during the task (stability was highest in the SMT, followed by naturalistic movie watching task and resting state). Next, we explored aging specific modulations in temporal stability matrices of dFC patterns between resting state and task and showed an overall increase in stability during the tasks across lifespan aging. To gather insight at each stage of adulthood, we divide *n* = 645 participants into 3 age categories—young (18–40 years), middle (41–60 years), and old adults (61–88 years). Our results revealed significant differences in temporal stability during task between young, middle, and old adults. Temporal stability was highest in young adults, followed by middle and old adults during both movie-watching and SMT. Applying our unsupervised method on specific resting-state brain networks revealed significant differences in temporal stability variation with age and allowed us to identify which of the resting-state subnetworks shape the global age-related pattern change. Finally, we examined the stochastic properties of temporal stability matrices using an auto-regressive modeling, and showed dominant whole-brain FC configurations are neither random nor Markovian. With our results, using a novel unsupervised method, we were able to demonstrate significant differences in stability of dFCs among conditions and age groups, which establishes the validity of our analysis—in the sense that low-dimensional dFC subspaces and subsequent estimation of temporal stability using quantitative metrics substantiated group differences and condition specific effects. We discuss the implications of these key results in the following subsections.

### Stochastic properties of dynamic functional connectivity

Studies describing brain dynamics have clustered recurring connectivity patterns into states, using clustering algorithms like K-means clustering (Allen et al. 2014; Damaraju et al. 2014; Cabral et al. 2017), HMM (Vidaurre et al. 2016; Cabral et al. 2017; Vidaurre et al. 2017; Quinn et al. 2018), suggestive of stability of functional architecture of the brain. Yet, most of the studies hypothesize a fixed number of discrete recurrent connectivity patterns or states with varying temporal fractional occupancy. The homogenous states are essentially clustered ignoring their temporal order and index. Studies have shown clustering time series requires ignoring some data and few attempts at clustering time series have shown to be objectively incorrect in some cases (Rakthanmanon et al. 2011; Rahman et al. 2020). Rahman and colleagues (Rahman et al. 2020) have proposed a novel framework, relying on the concept of shapelets, “statelets”—a high-dimensional state-shape representation of temporal dynamics of functional connectivity, instead of clustering. Another set of prior studies have explored the other side of stability - flexibility, which characterises heterogenous connectivity between a specific region and others over time (Yin, et al., 2016; Harlalka, Bapi, Vinod, & Roy, 2019) and temporal variability (Zhang & et al, 2016; Li, Lu, & Yan, 2019) of functional architecture in resting state (Li, Lu, & Yan, 2019), naturalistic movie watching task (Li, Lu, & Yan, 2019) and in disease (Zhang & et al, 2016). But these studies are restricted to temporal variability and flexibility of the functional architecture of a specific region. Our main contribution in this study is an unsupervised, data-driven approach to characterize the stability of whole-brain functional connectivity patterns. A recent study (Faghiri et al. 2020) has proposed a new method, where they calculate the gradients of timeseries pair and use their weighted average of shared trajectory (WAST) as a new estimator of dFC. This method defines a subspace on the raw BOLD fMRI timeseries where as our approach estimated dFC with BOLD phase coherence and defined dominant whole-brain FC patterns as dominant dFC subspaces with PCA and characterized temporally similar dominant whole-brain FC patterns with 2 alternative measures, angular distance and verifying the same with Mahalanobis distance (Fig. 1B). The central idea is if the dominant FC configurations are similar for extended time points, then they are considered to be stable.

Viduarre and colleagues (Vidaurre et al. 2017) have shown dynamic switching between brain networks and time spent visiting distinct brain networks are not random. Subsequently, another study has shown that the switching dynamics of functional brain states in the resting state follows AR model of order 1, or in other words a Markovian process fully explains the dFC evolution when correlation was computed using a sliding window approach (Liégeois et al. 2017). By constructing the unsupervised temporal stability matrices from 2 alternative approaches—principal angle, $\phi\ (t)$and Mahalanobis distance, $M(t)$, we reveal that dFC evolution is neither random nor Markovian (Fig. 7A and B) (Fig. 7C and D).

### Temporal stability of task-related dynamic functional connectivity is higher than rest

A key finding of our study indicates a global spread of shorter-lived, repeated patterns of stability between dominant FC configurations in resting state and local spread of longer-lived repeated patterns of stability in the task (in both continuous, naturalistic movie watching task and discrete goal oriented SMT) (Figs 2B and 3A). We find that in resting state the stability patterns are global, widespread with both short range (stability estimated between dominant dFC subspaces at timepoints in close proximity, for e.g.: ${\boldsymbol{D}}_{t_x}$at *t* = 15 and ${\boldsymbol{D}}_{t_y}\ \mathrm{at}\ t=25$) and long-range temporal stability patterns (stability estimated between dominant dFC subspaces at timepoints distant from one another, for e.g.: ${\boldsymbol{D}}_{t_x}$ at *t* = 15 and ${\boldsymbol{D}}_{t_y}$ at *t* = 150), whereas in task *temporal stability matrices,* the stability patterns are limited with fewer long-range patterns. The resting state is shown to be a multistable stationary state-regime at equilibrium. In the absence of any stimuli, the spontaneous resting state activity and the dynamics of formation and dissolution of RSNs form multiple stable states (Deco and Jirsa 2012). Ghosh and colleagues (Ghosh et al. 2008) have demonstrated that RSNs operate close to instability and explore these states before committing to one of these states. Deco and Jirsa (Deco and Jirsa 2012) have proposed that a repertoire of multistable states exists in resting state, which are functionally meaningful and inherently supported by the neuroanatomical connectivity, and can be rapidly activated even in the absence of any task. We speculate that in resting state the global spread of shorter-lived repeated patterns of stability between dominant FC configurations is associated with the exploration of multistable dynamic repertoire of states. On the contrary during a task (continuous or discrete), the repertoire of multistable states are limited, as only task-specific, cognitively relevant brain networks are explored. The brain visits task-specific stable states for duration that a putative stimulus triggered cognitive process demands. This is associated with the local spread of longer-lived temporal similarities between dominant functional connectivity subspaces in a task.

Our entropy results indicate the stability of functional connectivity architecture was highest in the discrete, goal-oriented SMT, followed by continuous naturalistic movie watching task and resting state (Fig. 4A). This is in line with previous studies, which report an increase in overall stability of FC with the largest increase in between-network connections (Elton and Gao 2015; Gonzalez-Castillo and Bandettini 2018), increase in stability of hemispheric homotopic connections during a task (Gonzalez-Castillo et al. 2014). Such increased stability of FC during a task is hypothesized to be associated with cognitive constraints during a task (Gonzalez-Castillo and Bandettini 2018). Frobenius distance analysis results reveal the temporal stability matrices of functional connectivity during continuous, naturalistic movie watching task was closer to resting state than discrete, goal-oriented SMT (Fig. 4C). Considering our Frobenius distance analysis, we hypothesized stability of functional connectivity architecture should be highest in the SMT, followed by the naturalistic movie watching task, which was validated by our entropy results. Our findings thus provide evidence of increased temporal stability of whole-brain functional connectivity in task, highest in the discrete, goal-oriented task, followed by continuous, naturalistic movie watching task and then resting-state, using a novel unsupervised approach of characterizing the stability of functional connectivity architecture.

### Aging introduces temporal variability in evolution of dynamic functional connectivity in both rest and task

Evidence from prior studies reveal the complexity of FC dynamics remains similar for all participants irrespective of age. An earlier study (Viviano et al. 2017) found no association between age and rate of switching between the FC states for resting brain. Our results (Figs 2B and 3A) indicate the overall trend of global spread of shorter-lived repeated patterns of stability between dominant FC configurations in resting state and local spread of longer-lived repeated patterns of stability in the task, was similar in young, middle, and old adults. Our study also revealed an overall trend of highest stability of functional connectivity in the discrete, goal-oriented SMT, followed by continuous, naturalistic movie watching task and resting state across lifespan aging (Fig. 4A). The neural noise hypothesis suggests the age-related cognitive decline could be explained as a consequence of the increase in the noisy baseline activity of the brain (Davis et al. 2009; Voytek et al. 2015). Our results, which contrasted the stability of functional architecture in young, middle, and old adults (Fig. 4B) found increased stability of functional architecture in young adults, followed by middle and old adults in movie watching and SMT. In accordance to neural noise hypothesis, the decrease in stability of the functional architecture of the brain in older adults can be explained with an increase in neural noise with age. Interestingly, McIntosh and colleagues (McIntosh et al. 2010) have reported BOLD signal variability of hub-region decreases with age, suggestive of increase in stability of hub regions with age. Our results, contrasting the stability of functional architecture of resting state in young, middle and old adults, report high stability in young and old adults and low stability in middle adults (Fig. 4B). Previous resting-state studies have reported quadratic or U-shaped trajectories of between-network connections with age (Betzel et al. 2014; Cao et al. 2014). Kupis et al. (2021) demonstrated a U-shaped or quadratic trajectory among between-network connections in certain resting-state brain states in relation with cognitive flexibility across lifespan aging. They also report shorter dwell time in middle adulthood and longer dwell time in childhood and older adulthood. Our result, scatter plot of entropy of whole-brain temporal dynamics matrices of resting-state across lifespan aging (*n* = 645 participants) (Fig. 5A) revealed an inverted U-shaped trajectory—with peak entropy among middle adults and low entropy among young and older adults. Our results are in line with (Kupis et al. 2021) as shorter dwell time in middle adulthood among brain states suggests decrease in stability of functional architecture in middle adults.

### Dynamics of whole brain resting state is primarily influenced by stability of sensorimotor network across lifespan aging

Earlier studies investigating age-related changes in RSN across lifespan have reported decrease in functional connectivity within DMN from early to late adulthood (Tomasi and Volkow 2012; Jockwitz and Caspers 2021). Betzel et al. (2014) found decrease in RSN modularity with age indicative of decrease in functional cohesiveness. Chen et al. (2021) reported, in older adults, decrease in dwell time on a state where DMN and attention networks show antagonistic activity. They report increase in dwell time on a “baseline” state, and, higher transition probabilities from others to this state in the elderly. Our results, using angular distance metric, demonstrate significant decrease in temporal stability of CEN and, visual networks in older adults (Fig. 5C and D). Whereas, the temporal stability of whole-brain resting state dFC and SM significantly increased with age (Fig. 5A and B). Of the 5 RSNs, we find stability dynamics of whole-brain resting state closely follows that of SM with age. Recently, Kong et al. (2021) have shown causal manipulation of a large-scale circuit model describing resting state brain dynamics, suggests that sensorimotor regions are a driver of FC dynamics. King et al. (2017) attribute age-related decline in motor performance to decrease in segregation of large-scale brain networks rather than age-related connectivity changes within motor-related network. Our results demonstrate the influence of stability of SM on stability dynamics of whole-brain resting state, which (King et al. 2017) have associated with age-related decline in motor performance. We did not find significant differences in temporal stability of RSNs with age using Mahalanobis distance metric (see Supplementary S7). The differences in temporal stability of dominant subspaces across RSNs with angular distance and Mahalanobis distance could be an outcome of the limitations of computing angular/Mahalanobis distance between linear subspaces. The Mahalanobis distance is a multidimensional generalization of the distance between a point P and a distribution D (Mahalanobis 1930). In the current study, for each timepoint, Mahalanobis distance was calculated between each ROI in the reduced dominant dFC subspace ${\boldsymbol{D}}_{t_y}$and whole-brain subspace ${\boldsymbol{D}}_{t_x}$. Every ROI in ${\boldsymbol{D}}_{t_y}$ (point P) has Mahalanobis distance estimated with respect to subspace ${\boldsymbol{D}}_{t_x}$ (distribution D). In our case, Mahalanobis distance is the mean of the distance of all the points (i.e. ROIs) in dominant dFC subspace at *t* = ${t}_i$ with distribution of dominant dFC subspace at *t* = ${t}_j$, whereas angular distance is the scalar angle between the dominant dFC subspace at *t* = ${t}_i$ with dominant dFC subspace at *t* = ${t}_j$. Mahalanobis distance takes into account the correlations of the data, and is a scale-invariant measure (De Maesschalck et al. 2000). Another reason may be due to the high dimensionality of the dFC, the data are sparsely distributed in the high-dimensional subspace, referred to as “curse of dimensionality” (Kuo and Sloan 2005). Although PCA is a robust dimensionality reduction technique, used to circumvent “curse of dimensionality,” studies have found PCA is sensitive to outliers (Candes et al. 2011), and degradation of performance of PCA when the dimensionality of data increases (Shetta and Niranjan 2020).

### Limitations and future directions

An important caveat of the current study was due to parcellation atlas used in the Cam-CAN dataset. The AAL atlas parcellates the brain regions into 116 structural parcels and few parcels span multiple functional regions. For future studies, for a more refined spatial profile of temporal stability of functional architecture, using a finely parcellated brain atlas is recommended. Researchers have shown stability of functional architecture is modified in patients of Schizophrenia, ADHD and ASD (Zhang et al. 2016; Guo et al. 2017). Hence, we can extrapolate that the temporal stability of functional architecture can provide a richer information to discover biomarkers for neurological and mental disorders. A meaningful extension of the present work would be to investigate the influence of subject-level characteristics and even, behavioral scores on temporal stability of FC. We want to address this in a future study including all different variables and demographic information over adult life span. This exercise could also strengthen and provide evidence for the robustness of the method and may aide in uncovering temporal stability markers specific to the tasks.

## Conclusion

In summary, the current study introduces a data-driven unsupervised approach to characterize the temporal stability of functional architecture. When applied to a putative lifespan aging data, the whole-brain temporal dynamics of naturalistic movie watching task was found to be closer to resting state than during SMT. Further, the study revealed peak temporal stability in SMT, followed by naturalistic movie watching task and resting state, a trend similar in both young, middle, and elderly. The dynamics of the temporal stability of functional architecture of the whole-brain resting state was primarily influenced by temporal stability of SM across lifespan aging. The quantification of differences in network stability associated with healthy aging provides evidence for the potency of the temporal stability measure to act as biomarker for multiple neurological disorders.

**Abbreviations**
FC: functional connectivity,
DMN: default mode network
CEN: central executive network
SM: sensorimotor network
AAL: automated anatomic labelling atlas

## Supplementary Material

SupplementaryDocument_bhac133

## Data/Code availability statement

The datasets generated during the analysis pipelines in the present study are available from the corresponding author on reasonable request. The codes for all the analysis carried out in this paper are available on https://bitbucket.org/cbdl/workspace/projects/DFC.
