# Supplementary material for: Stability of sensorimotor network sculpts the dynamic repertoire of resting state over lifespan
Source: Cereb Cortex. 2022 Apr 4;33(4):1246–62. doi: 10.1093/cercor/bhac133 (PMC9930636; doi:10.1093/cercor/bhac133)
Supplement: SupplementaryDocument_bhac133 [file supplementarydocument_bhac133.docx]

**Supplementary figures**

**S1**


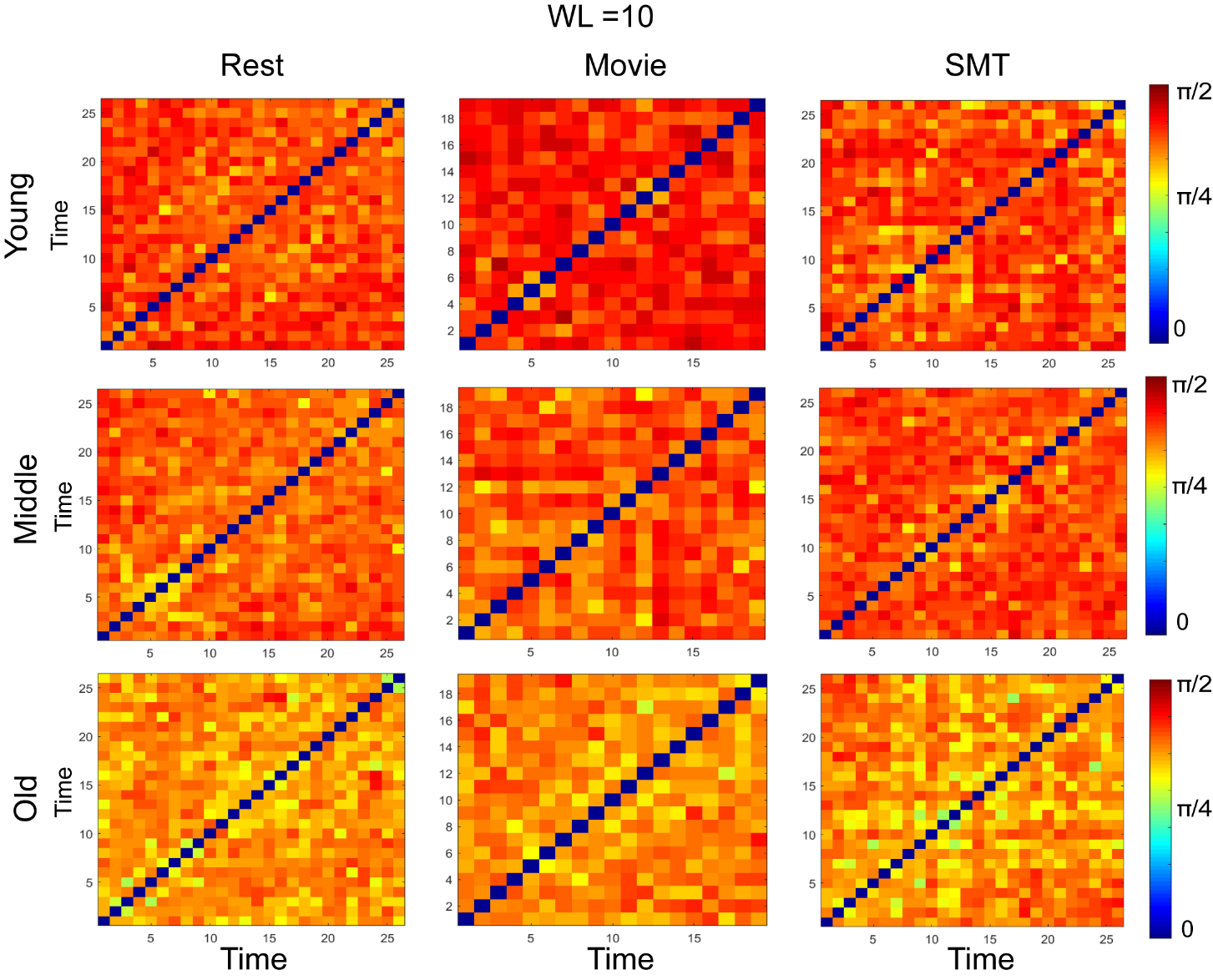


**S1:** Temporal stability matrices of the resting state, naturalistic movie watching task and sensorimotor task, where each entry is the principal angle $\phi(t_{x},t_{y})$ between dominant dFC subspaces at $t_{x}$ and $t_{y}$, for young and old adults. For validation of the results where dFC was estimated using BOLD phase coherence, we calculated dFC using sliding window approach with (window length) WL = 10 time points.


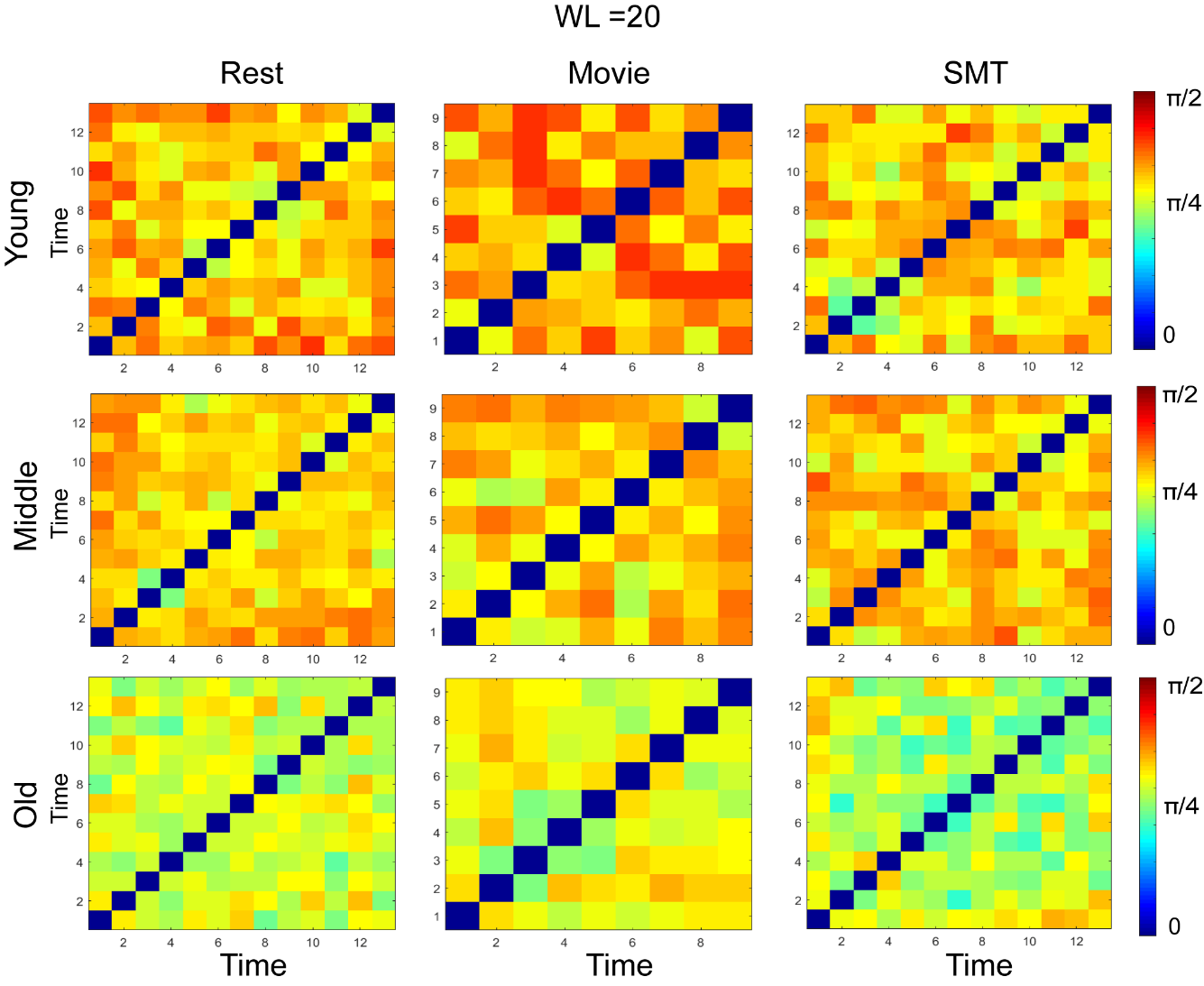


**S2**

**S2**: Temporal stability matrices of the resting state, naturalistic movie watching task, and sensorimotor task, for both young and old adults. dFC was estimated using sliding window approach with (window length) WL= 20 time points.

**S3**


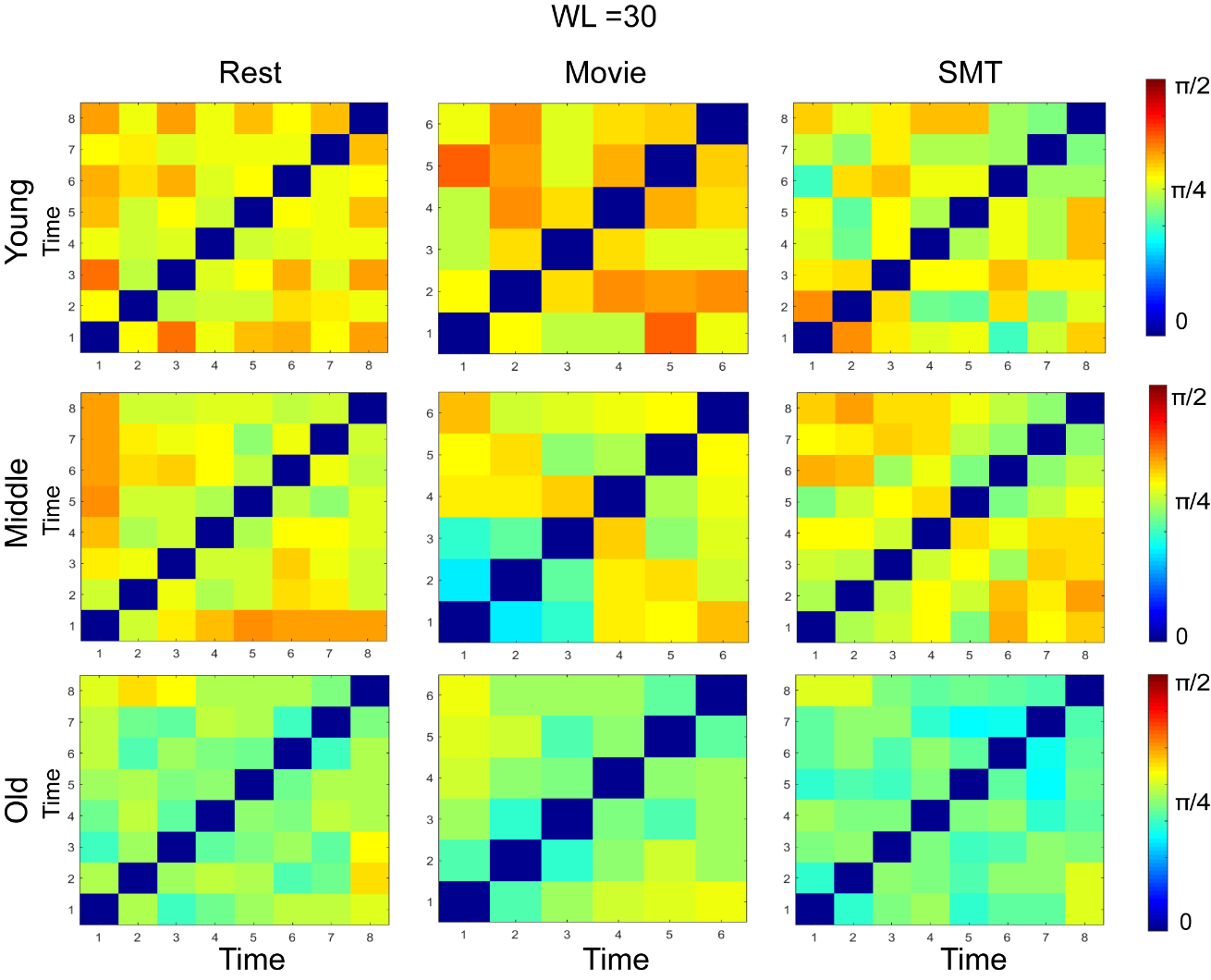


**S3:** Temporal stability matrices of the resting state, naturalistic movie watching task, and sensorimotor task, for both young and old adults. dFC was estimated using sliding window approach with (window length) WL= 30 time points

**S4**


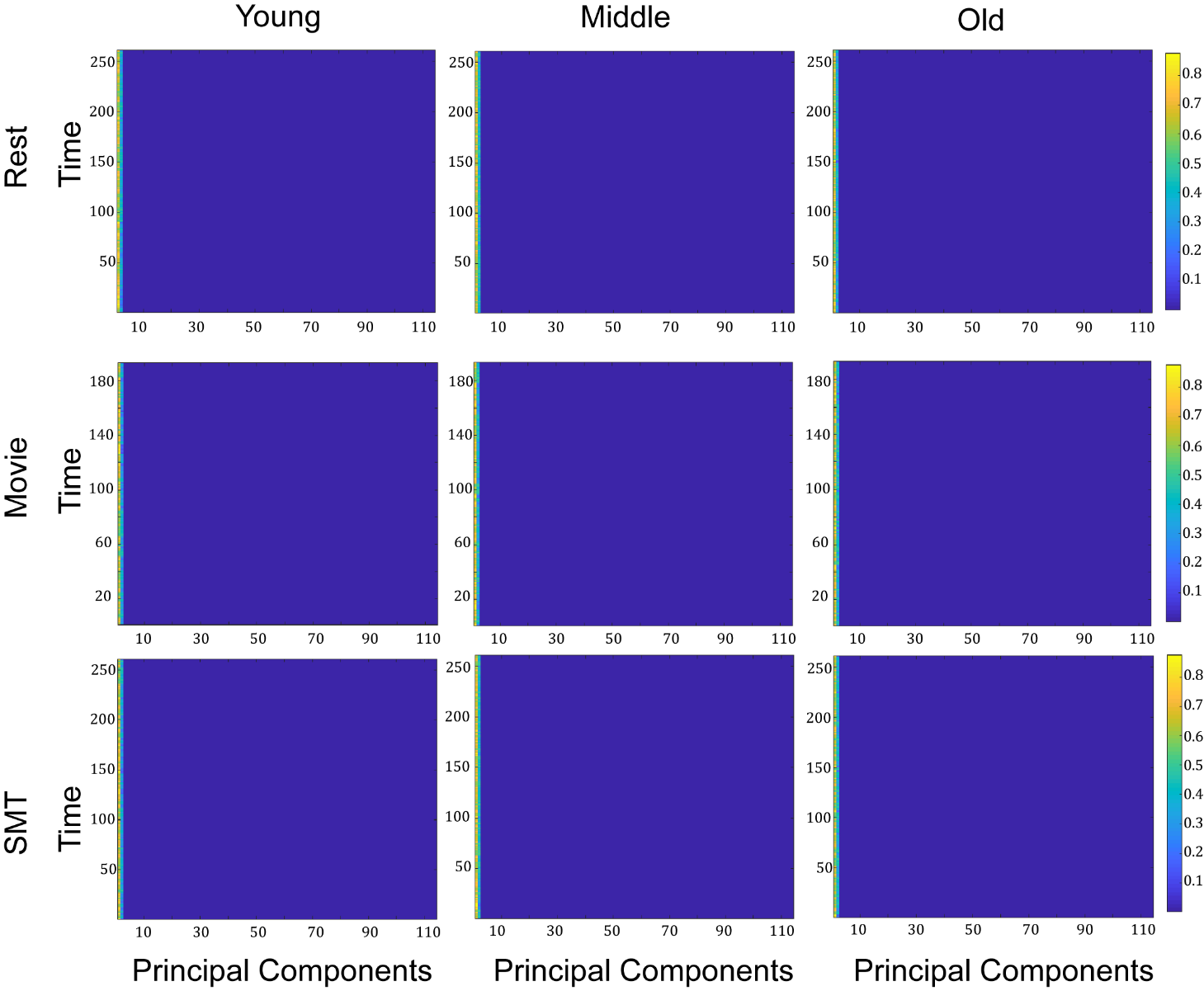


**S4:** The Plot represents the variance explained by all 116 principal components of the input dFC matrix for all categories. The first three principal components explain almost 99% of the variance of the input matrix.


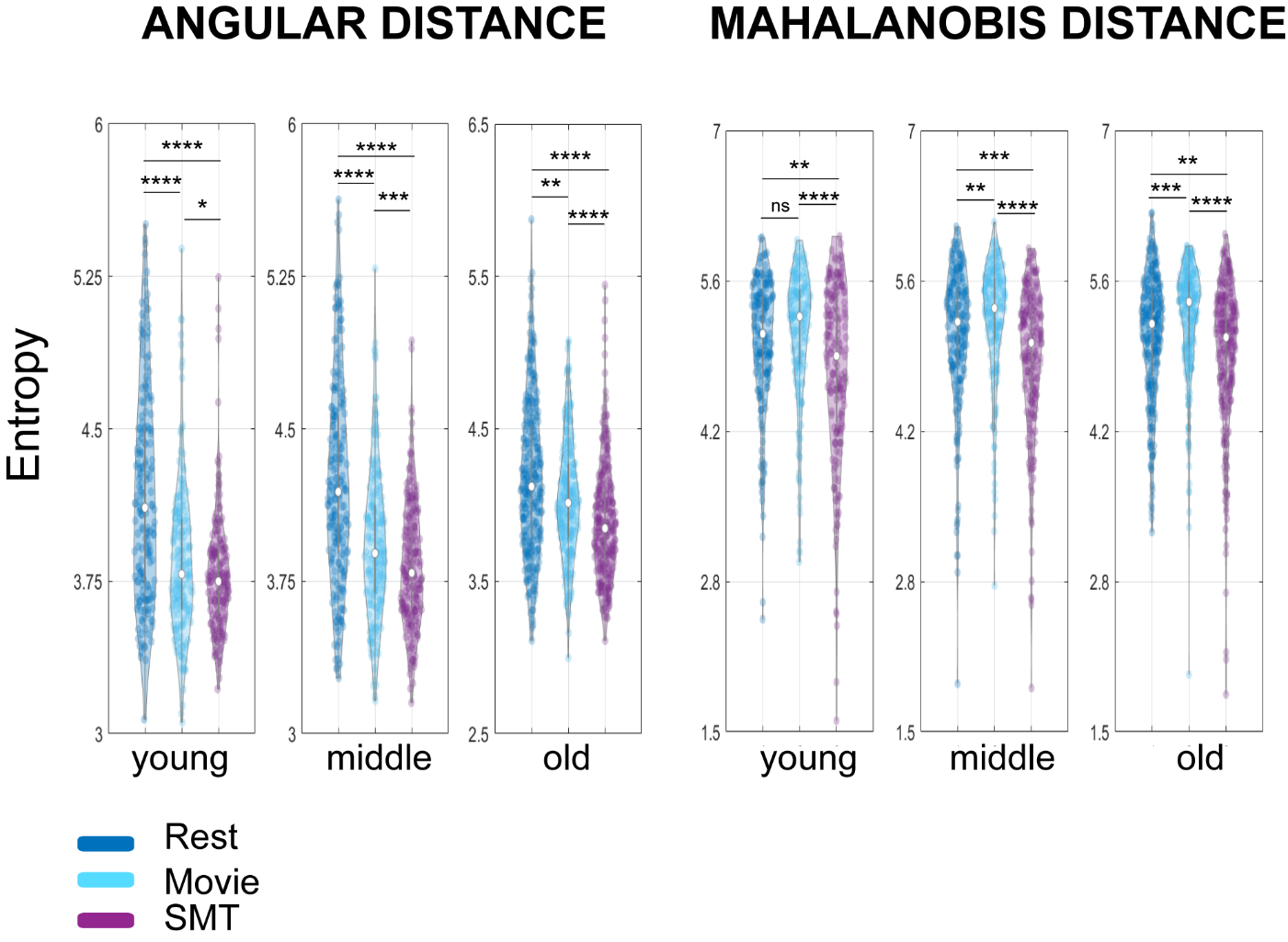
**S5**

**S5:** The plot represents entropy of temporal stability matrices of resting state (rest), movie watching (movie) and sensorimotor task (smt) across young, middle and old adults, in both angular distance and Mahalanobis distance metric.

In angular distance, comparing the median values, peak entropy was reported in temporal stability matrices of resting state, followed by movie watching task and sensorimotor task. The distributions were non-parametric. Wilcoxon sign rank test revealed significant differences between entropy of temporal dynamics matrices of resting state and movie watching task, movie watching and sensorimotor task and sensorimotor task and resting state in young, middle and old adults as shown below:

| **Angular distance** | **rest - movie** | **movie-smt** | **smt-rest** | |
| --- | --- | --- | --- | --- |
| Young | p=1.10e-10 | p=0.034 | p=2.6e-17 |  |
| Middle | p=6.9e-11 | p=5.19e-04 | p=5.65e-21 |  |
| Old | p=0.0013 | p=4.9e-09 | p=9.81e-17 |  |

In mahalanobis distance, although the median values report peak entropy in movie watching task, followed by resting state and sensorimotor task, the violin plot indicates high variability of entropy values in resting state and movie watching task among middle and elderly.

Wilcoxon sign rank test revealed significant differences between entropy of temporal dynamics matrices of resting state and movie watching task, movie watching and sensorimotor task and sensorimotor task and resting state in young, middle and old adults as shown below:

| **Mahalanobis distance** | **rest - movie** | **movie - smt** | **rest - smt** | |
| --- | --- | --- | --- | --- |
| Young | **ns** | p=1.9e-05 | | p=0.0014 |
| Middle | p=0.0094 | p=1.65e-10 | | p=2.9e-04 |
| Old | p=7.6e-04 | p=1.7e-10 | | p=0.0073 |

**S6**


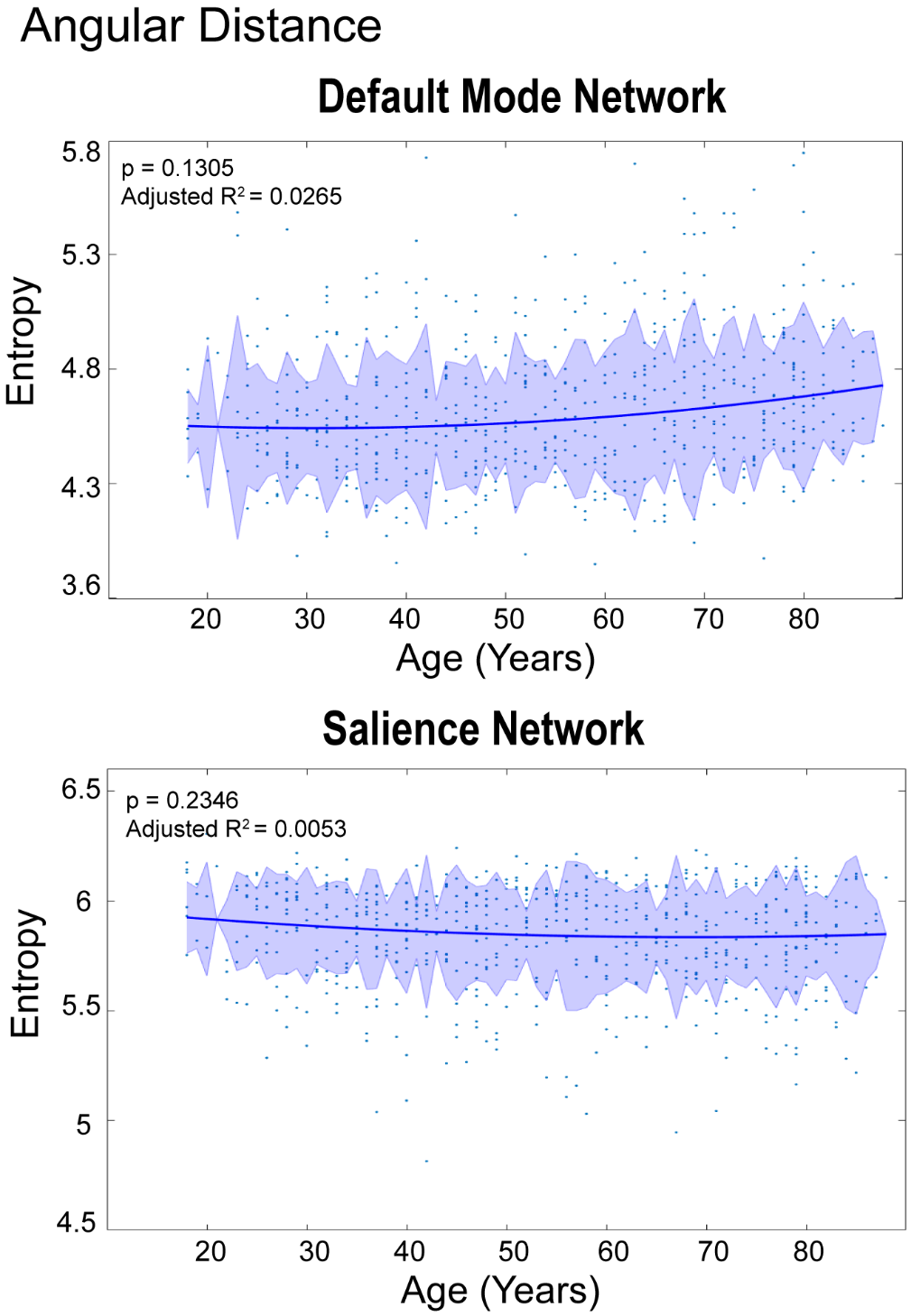


**S6:** Scatter plot of entropy of temporal dynamics matrices of resting state brain network subspaces (default mode network, salience network) across lifespan ageing (N=645 participants) estimated with angular distance metric. A quadratic regression model is fitted onto the entropy values. ‘p’ is effectively a 2-sided t-test against the corresponding coefficient being zero.

**S7**


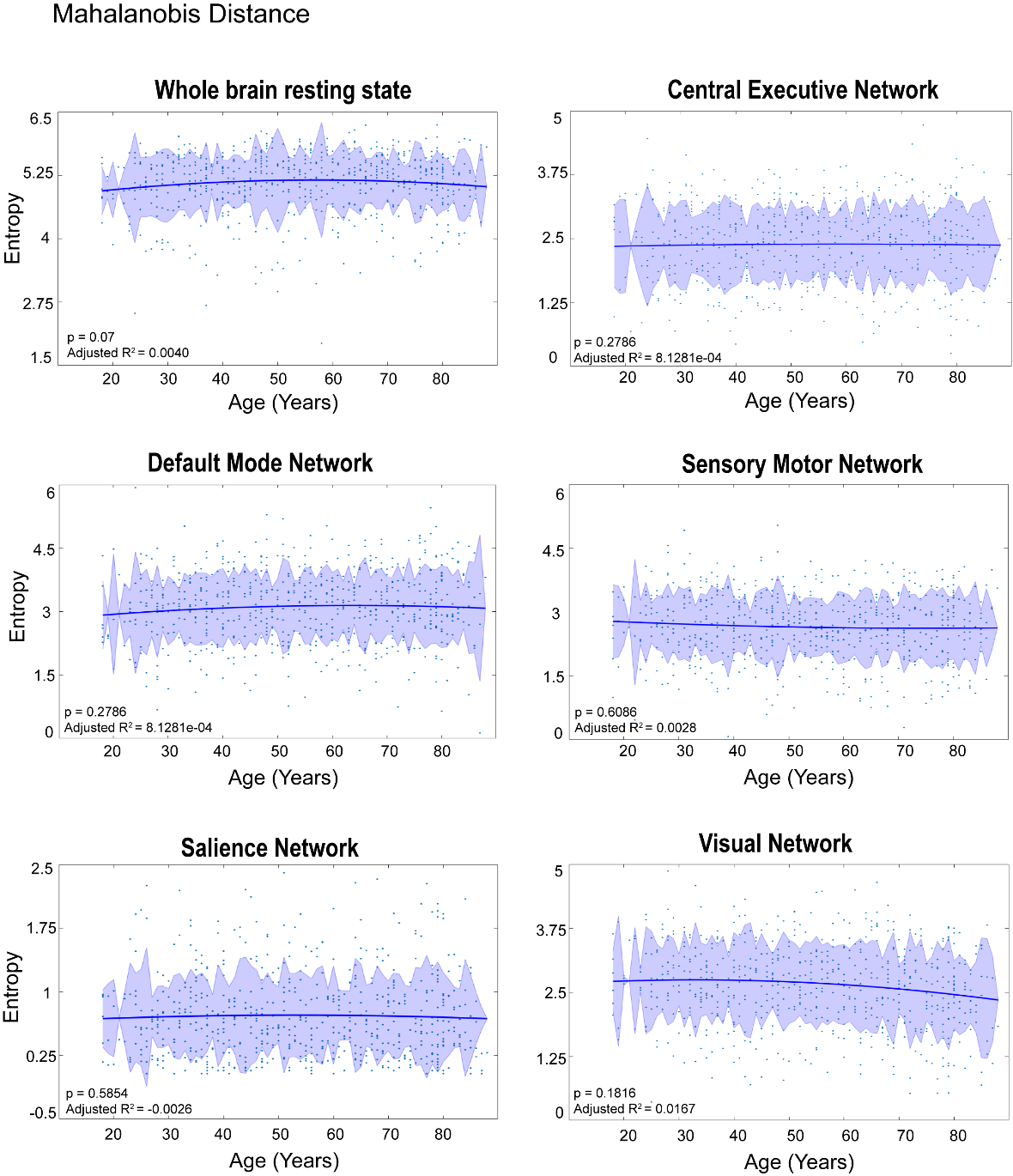


**S7:** Life span ageing related changes in temporal stability of dynamic functional connectivity subspace of whole-brain resting state, central executive network, default mode network, sensorimotor network, salience network and Visual network estimated with Mahalanobis distance metric. A quadratic regression model is fitted onto the entropy values. ‘p’ is effectively a 2-sided t-test against the corresponding coefficient being zero.

**S8**

**Angular Distance:**

| **Networks** | **Co-efficient** | **p** | $\boldsymbol{R}^{\boldsymbol{2}}$ | **Adjusted**  $\boldsymbol{R}^{\boldsymbol{2}}$ | **Equation** |
| --- | --- | --- | --- | --- | --- |
| CEN | [1.1114e-04, -0.0095, 5.2476] | [ 6.5e-04,0.0073, 1.2875e-262] | 0.0460 | 0.0430 | $1 .1{{\left( e-4 \right)X}_{1}}^{2}- 9.5\left( e-3 \right)X_{1} + 5.2$ |
| DMN | [5.7274e-05, -0.0035, 4.6379] | [0.1305, 0.3997, 1.7487e-200] | 0.0295 | 0.0265 | $5.7{{\left( e-5 \right)X}_{1}}^{2}- 3.5\left( e-3 \right)X_{1} + 4.6$ |
| Salience  network | [3.5140e-05 -0.0048 5.9851] | [0.2349 0.1366 2.3768e-317] | 0.0084 | 0.0053 | $3 .5{{\left( e-5 \right)X}_{1}}^{2}- 4.8\left( e-3 \right)X_{1} + 6.0$ |
| Sensorimotor  Network | [-1.2182e-04 0.0123 4.7931] | [0.0104 0.0177 8.9857e-162] | 0.0118 | 0.0087 | $-1 .2{{\left( e-4 \right)X}_{1}}^{2}+ 0.012X_{1}+$  $4.8$ |
| Visual  Network | [1.1163e-04 -0.0121 5.3007] | [0.0068 0.0069 1.8304e-211] | 0.0114 | 0.0083 | $1 .1{{\left( e-4 \right)X}_{1}}^{2}- 0.012X_{1} + 5.3$ |
| Whole brain  resting state | [-1.5515e-04 0.0161 3.8349] | [0.0136, 0.0188 1.0251e-82] | 0.0099 | 0.0068 | $-1 .6{{\left( e-4 \right)X}_{1}}^{2}+ 0.016X_{1} +$  $3.8$ |

**Mahalanobis Distance:**

| **Networks** | **Co-efficient** | **p** | $\boldsymbol{R}^{\boldsymbol{2}}$ | **Adjusted**  $\boldsymbol{R}^{\boldsymbol{2}}$ | **Equation** |
| --- | --- | --- | --- | --- | --- |
| CEN | [-2.6738e-05 0.0032 2.3478] | [0.7775 0.7539 1.0861e-18] | 1.99e-04 | -0.0029 | $-2.7{{\left( e-5 \right)X}_{1}}^{2}+ 3.2\left( e-3 \right)X_{1} + 2.3$ |
| DMN | [- 1.1493e-04 0.0145 2.7252] | [0.2786 0.2105 7.4536e-20] | 0.0039 | 8.1281e-04 | $-1 .4{{\left( e-4 \right)X}_{1}}^{2}+ 0.014X_{1} + 2.7$ |
| Salience  network | [-3.3604e-05 0.0036 0.6337] | [0.5854 0.5962 1.7579e-04] | 4.68e-04 | -0.0026 | $-3 .4{{\left( e-5 \right)X}_{1}}^{2}+ 3.6\left( e-3 \right)X_{1} + 0.63$ |
| Sensorimotor  network | [ 5.0499e-05 -0.0075 2.9144] | [0.6086 0.4836 2.8610e-25] | 0.0028 | -3.0774e-04 | $5.5{{\left( e-5 \right)X}_{1}}^{2}- 7.5\left( e-3 \right)X_{1} + 2.9$ |
| Visual  network | [-1.3325e-04 0.0086 2.5903] | [0.1816 0.4264 3.1123e-20] | 0.0198 | 0.0167 | $-1.3{{\left( e-4 \right)X}_{1}}^{2}+ 8.6\left( e-3 \right)X_{1} +2.6$ |
| Whole brain  resting state | [-1.3750e-04 0.0163 4.6653] | [0.0700 0.0490 1.4997e-83] | 0.0071 | 0.0040 | $-1.4{{\left( e-4 \right)X}_{1}}^{2}+0.016X_{1} + 4.7$ |

**S8:** Detailed overview of the terms included in the regression analysis (**Figure 5** and supplementary figures **S6** and **S7.** We use Polyfitn function directory by John D’Errico (John D’Errico (2022). polyfitn (https://www.mathworks.com/matlabcentral/fileexchange/34765-polyfitn), MATLAB Central File Exchange. Retrieved January 12, 2022.) for our regression analysis. The details of the terms included in the analysis are tabulated below. ***p*** is effectively a 2-sided t-test against the corresponding coefficient being zero. Adjusted R^2 accounts for changing degrees of freedom in the model

**Addendum:**

**Fig 1** in colour:


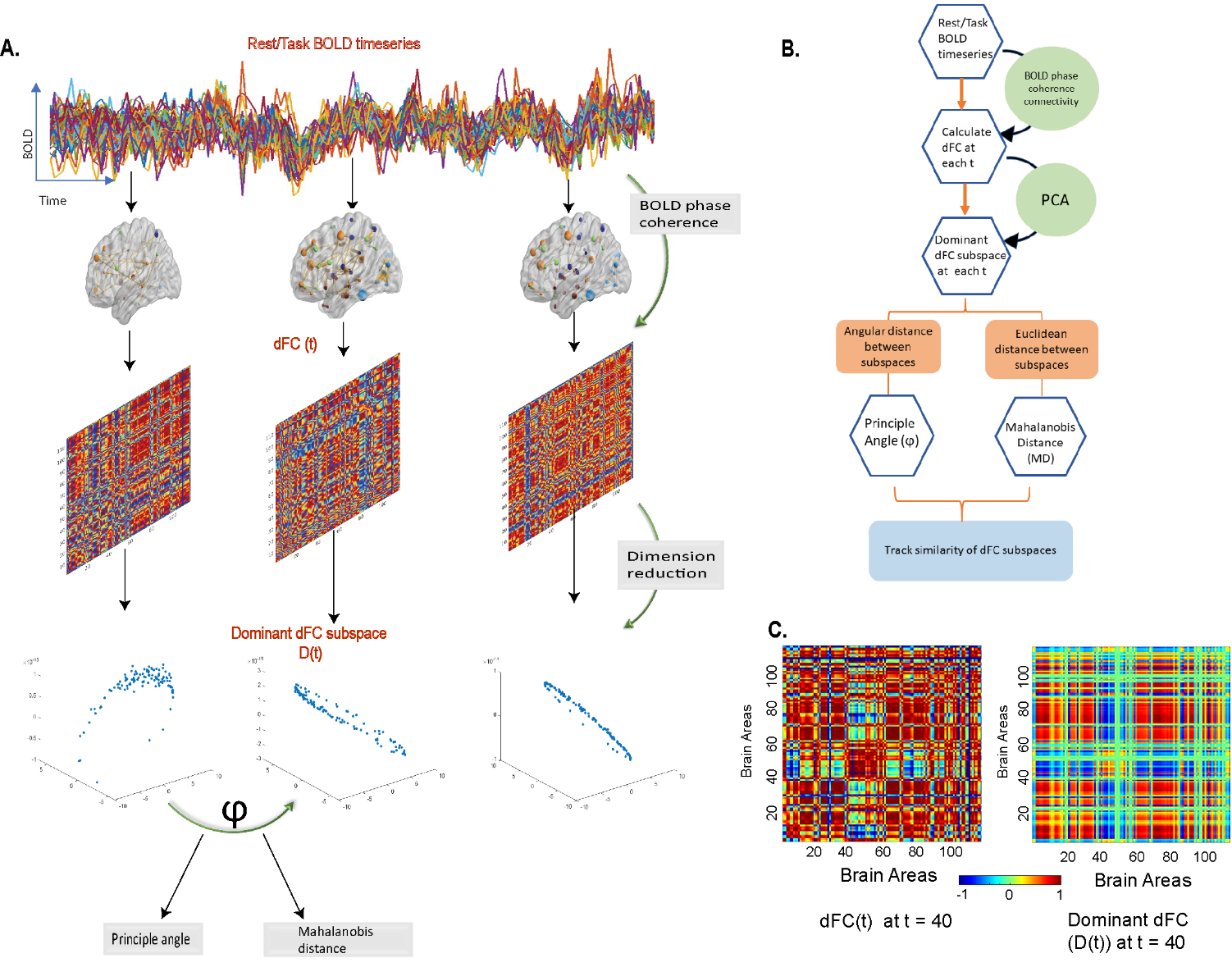


**Figure 1:** *Brief overview of the unsupervised approach* ***(A)****. The schematic diagram shows how the temporal stability of dynamic functional connectivity subspaces (dFC) are computed. Dominant dFC subspace, at each time point, is estimated using the first three principal components of dFC(t), that was computed using the measure of BOLD phase coherence. The similarity between dFC subspaces are calculated using Angular distance (principal angle) and Mahalanobis distance (Euclidean distance). If the dominant dFC subspaces are similar for extended timepoints, then they are considered to be stable.* ***(B).*** *A flowchart representation of the method* ***(C).*** *Matrix representation of dFC patterns* ***(dFC(t))*** *and reduced Dominant dFC patterns* ***(D(t))*** *at t=40.*

**Fig 2** in colour:


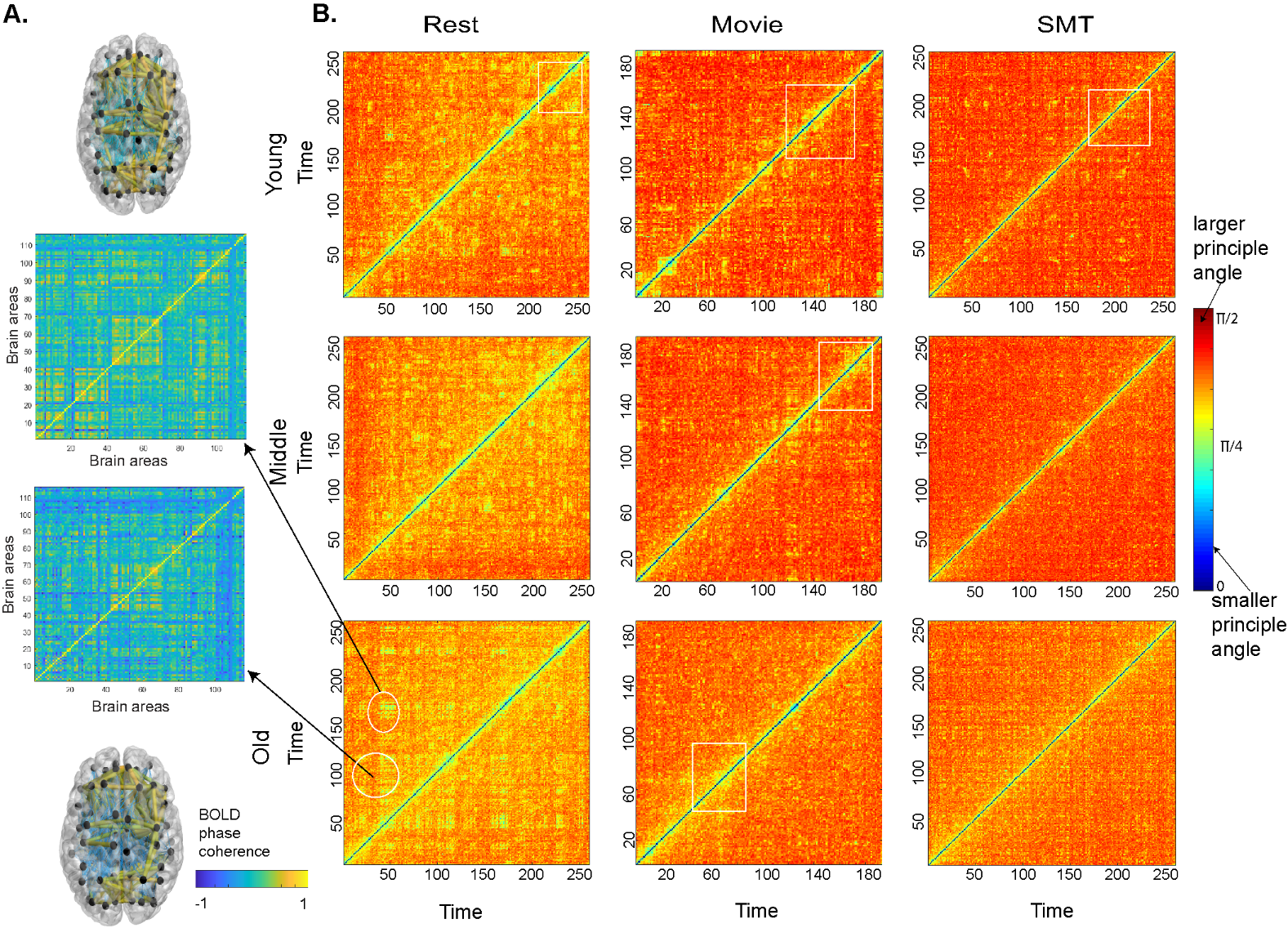


**Figure 2*:*** *Using angular distance to characterise temporal stability matrices across age* ***(A)*** *dFC matrices estimated using BOLD phase coherence.* ***(B)*** *Time X Time temporal stability matrix of resting state, naturalistic movie watching task and discrete, sensorimotor task for young, middle and old adults. Each entry in the matrix is the principal angle* $\phi(t_{x},t_{y})$ *between dominant dFC subspaces at* $t_{x}$ *and* $t_{y}$*. The principal angle ranges between 0 (low angular distance) to π/2 (high angular distance). Resting state, in young, middle and old adults, has shorter-lived, global spread of patterns of temporal stability. On the contrary, both the tasks have a longer-lived, local spread of patterns of stability (indicated by arrows and rectangular boxes).*

**Fig 3** in colour:


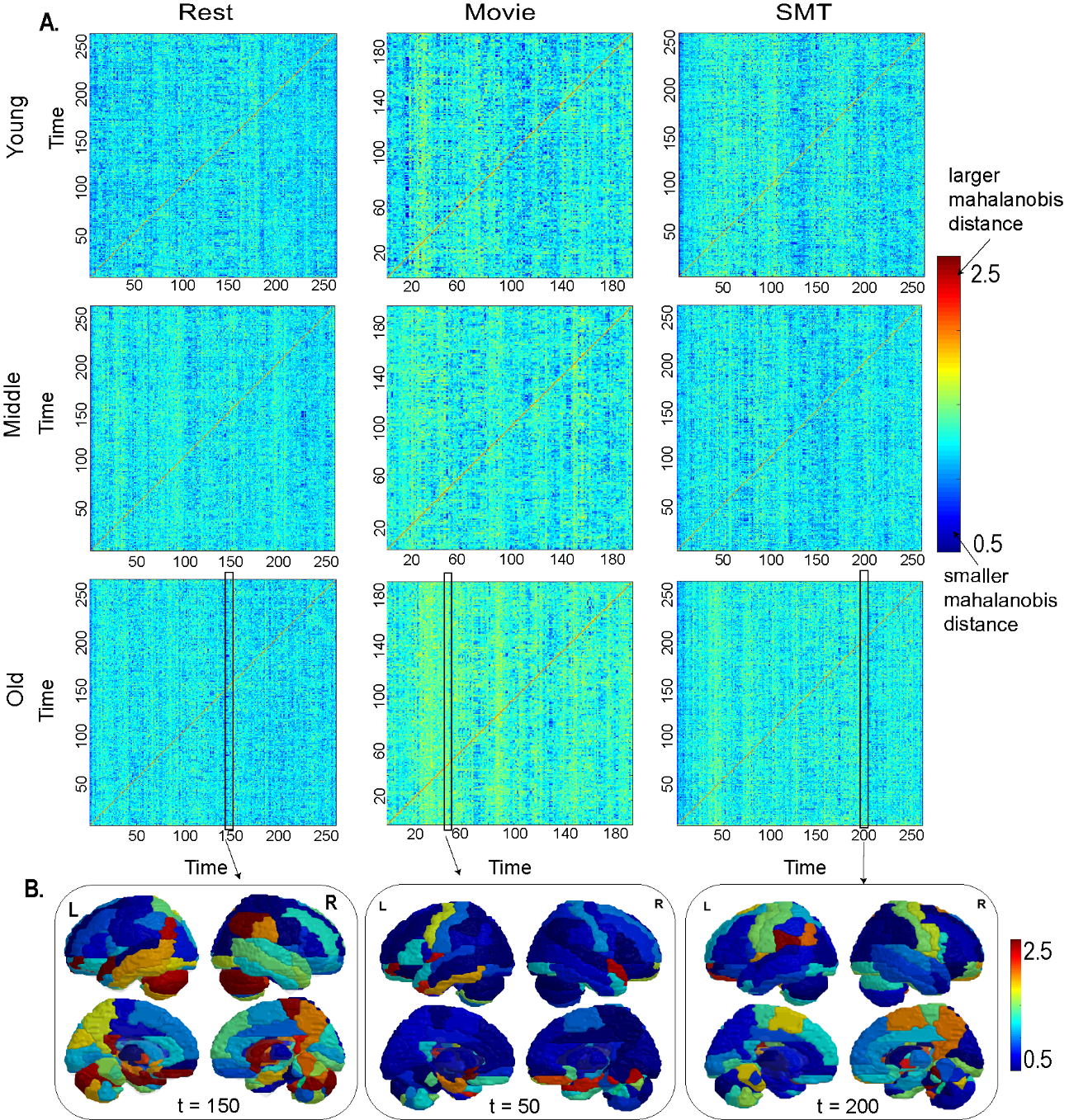


**Figure 3:** *Using Mahalanobis distance to characterise temporal stability matrices across age.* ***(A)*** *Time X Time temporal stability matrix of resting state, naturalistic movie watching task, and sensorimotor task for young, middle and old adults, where each entry in the matrix is Mahalanobis* $\left( M^{2}\left( t_{x},t_{y} \right) \right)$ *distance between the dominant dFC subspaces. Mahalanobis distance between dominant dFC subspaces is low when the dFC configurations are similar.* ***(B)*** *The profile of temporal stability estimated with Mahalanobis distance between dominant dFC subspaces at t=15 and t=150, t=50, t=200 across the brain regions.*
